# Fruit fly phylogeny imprints bacterial gut microbiota

**DOI:** 10.1111/eva.13352

**Published:** 2022-05-03

**Authors:** Virginie Ravigné, Nathalie Becker, François Massol, Erwan Guichoux, Christophe Boury, Frédéric Mahé, Benoit Facon

**Affiliations:** ^1^ CIRAD UMR PHIM Montpellier France; ^2^ PHIM Univ Montpellier CIRAD INRAE Institut Agro IRD Montpellier France; ^3^ 52827 UMR ISYEB MNHN Paris France; ^4^ Inserm CHU Lille Institut Pasteur de Lille U1019 – UMR 9017 Center for Infection and Immunity of Lille (CIIL) CNRS Université de Lille Lille France; ^5^ INRAE ‐ UMR 1202 BIOGECO ‐ Plateforme Genome Transcriptome de Bordeaux Cestas France; ^6^ 27057 UMR CBGP INRAE Montferrier‐sur‐Lez France

**Keywords:** community ecology, long‐read sequencing, metabarcoding

## Abstract

One promising avenue for reconciling the goals of crop production and ecosystem preservation consists in the manipulation of beneficial biotic interactions, such as between insects and microbes. Insect gut microbiota can affect host fitness by contributing to development, host immunity, nutrition, or behavior. However, the determinants of gut microbiota composition and structure, including host phylogeny and host ecology, remain poorly known. Here, we used a well‐studied community of eight sympatric fruit fly species to test the contributions of fly phylogeny, fly specialization, and fly sampling environment on the composition and structure of bacterial gut microbiota. Comprising both specialists and generalists, these species belong to five genera from to two tribes of the Tephritidae family. For each fly species, one field and one laboratory samples were studied. Bacterial inventories to the genus level were produced using 16S metabarcoding with the Oxford Nanopore Technology. Sample bacterial compositions were analyzed with recent network‐based clustering techniques. Whereas gut microbiota were dominated by the Enterobacteriaceae family in all samples, microbial profiles varied across samples, mainly in relation to fly identity and sampling environment. Alpha diversity varied across samples and was higher in the Dacinae tribe than in the Ceratitinae tribe. Network analyses allowed grouping samples according to their microbial profiles. The resulting groups were very congruent with fly phylogeny, with a significant modulation of sampling environment, and with a very low impact of fly specialization. Such a strong imprint of host phylogeny in sympatric fly species, some of which share much of their host plants, suggests important control of fruit flies on their gut microbiota through vertical transmission and/or intense filtering of environmental bacteria.

## 1 INTRODUCTION

Agro‐ecosystems comprise a significant proportion of land use and harbor a non‐negligible fraction of biodiversity (Pimentel et al., 1992; Tilman et al., 2011). More than many others, these ecosystems suffer from intense structural anthropogenic alterations. Conflicting imperatives to intensify production while simultaneously reducing environmental impacts increasingly drive short‐term and fine‐scale ecological and evolutionary processes (Thrall et al., 2011), demanding greater capacity to predict and manage their consequences (Gilligan, 2008). One promising avenue for reconciling the goals of crop production and ecosystem preservation consists in manipulating quantitatively and/or qualitatively beneficial biotic interactions (Gaba et al., 2014; Massol & Petit, 2013). Over the last decade, this strategy has taken a new turn by considering risks and opportunities associated with plant and insect microbiota. In particular, microbes associated with phytophagous insect are thought to offer great potential for improved management of economically important pests (Crotti et al., 2012). For instance, gut bacteria can be used to reverse radiation‐induced fitness decrease in sterile males used in the sterile insect technique, to produce new bacterial odoriferous attractants for insect traps, or to stimulate insect behaviors such as feeding or oviposition (Noman et al., 2020; Raza et al., 2020). Yet, identification of the associated microbial species, and of their respective role in plant–insect interactions and dynamics, is still far from complete.

There is now good agreement on the idea that microbes may play an important role in host adaptation (Macke et al., 2017). In particular, one of the major arenas for host–microbe interactions is the insect gut, which is typically colonized by a large number of diverse microbes, among which bacterial associations predominate (Engel & Moran, 2013). Empirical evidence accumulates, showing that insect gut microbiota can affect host fitness by contributing to development, host immunity, nutrition, or behavior (Kolodny et al., 2020). Gut microbes have even been suspected to be the hidden key player of plant exploitation by their insect pests, as, for example, for the olive fly *Bactrocera oleae* (Ben‐Yosef et al., 2014) and the coffee berry borer *Hypothenemus hampei* (Ceja‐Navarro et al., 2015).

Gut microbiota are complex, heterogeneous, and variable communities of microbes. First, they assemble within each host generation through different transmission routes. Specifically, gut microbes are mainly acquired via horizontal transfer from the surrounding environment (Broderick & Lemaitre, 2012). However, a number of mechanisms exist for inoculating progeny with microbial symbionts, increasing rates of vertical transmission, and enabling long‐term associations (Engel & Moran, 2013). For example, in some flies eggshells are contaminated with parental bacteria (Capuzzo et al., 2005; Raza et al., 2020). Even when acquired horizontally at each generation, gut communities are not random assemblages of bacteria from the food or local environment, due to host filtering and promoting specific bacteria (Engel & Moran, 2013). Second, insect species vary immensely in their dependence on gut microbes: Some almost lack them entirely, while others have developed obligate dependence (Moran et al., 2019). Third, host–microbiota interactions extend along the parasite–mutualist continuum and the exact position may change according to the cost–benefit balance resulting from interactions between bacteria composing the microbiota (Mushegian & Ebert, 2016). Fourth, gut microbiota are often considered as having a multilayered structure (Shapira, 2016). One layer would be the so‐called core microbiota, which would tend to be under host genetic and immune control, reliably transmitted across generations, and sharing evolutionary interests with the host (Macke et al., 2017). Some of these microbes may be beneficial to the host and contribute to essential functions or provide long‐term adaptation to stable features of the host niche (Nougué et al., 2015). A second layer would be composed of a flexible, environment‐modulated pool of microbes, varying within the course of individual life and exhibiting high interindividual variation. Because of possibly divergent evolutionary interests, microbes from this second layer could either be beneficial or detrimental to the host (Macke et al., 2017), potentially depending on the rest of the gut microbiota members (Mushegian & Ebert, 2016).

In relation to this important variability of insect–microbe associations, understanding the role of gut microbiota in plant–insect interactions may benefit from deciphering the determinants of gut microbiota composition and structure. Gut microbiota are affected by many factors, including host phylogeny and host ecology (Spor et al., 2011). First, the environment in which insects develop and live strongly determines the set of bacteria, with which they will have an opportunity to associate. In phytophagous insects, the environments encountered are not random. They depend on insect ecology, a major feature of which is host range, that is, the host plant species an insect uses. For instance, one could expect that insect species specialized on different host plants encounter different initial microbe pools and that generalist insect species encounter a more diverse set of microbes than specialist species (Deb et al., 2019). Second, host phylogeny could potentially structure insect gut microbiota through different mechanisms ranging from active filters (constrained by host development, immune function morphology, and physiology), to the sharing of similar microbe pools (through social interactions or similarity in diet; Brooks et al., 2016). While host phylogeny, host specialization, and sampling environment factors are all considered as potential determinants of gut microbial communities, their relative importance is still a matter of debate, not only because it probably varies across taxa but also because of the associated technical challenge. Studies generally compare gut microbiota among related host species with contrasting ecologies in natural environments (Ivens et al., 2018), and through broad phylogenetic sampling of animals with both divergent and convergent feeding ecologies (Nishida & Ochman, 2018). However, in addition to their differences in phylogenetic history and level of specialization, surveyed host species may differ in their geographic ranges, thus experiencing different microbial species pools in their local environment. Controlled or laboratory environments, used for studies of closely related host taxa (Erlandson et al., 2018; Kohl et al., 2018), may partially reduce this bias. However, sampled microbial pools are unlikely to be representative of those encountered in the wild. This limitation can be overcome by analyzing microbiota in sympatric species of known ecology and phylogenetic history (Martinson et al., 2017).

Reunion, a small island in South‐West Indian Ocean, harbors a community of eight sympatric fruit flies, considered as the main actors in the local guild of fruit‐eating phytophagous arthropods (Quilici & Jeuffrault, 2001), which could constitute a convenient system to tackle this question. These species belong to five genera from two tribes of the Tephritidae family (Moquet et al., 2021): Three species are Ceratitinae (*Ceratitis capitata*, *Ceratitis quilicii*, and *Neoceratitis cyanescens*), and five species are Dacinae (closely related species *Bactrocera dorsalis*, *Bactrocera zonata*, and *Zeugodacus cucurbitae* on the one hand, and *Dacus ciliatus* and *Dacus demmerezi*, on the other). They differ in their level of specialization: Four are generalist species (the *Ceratitis* and *Bactrocera* species, commonly found on more than 30 plant species of several distant plant families), three are specialists of Cucurbitaceae (the *Dacus* and *Zeugodacus* species), and one is a specialist of Solanaceae (*N*. *cyanescens*). Most importantly, both tribes comprise specialist and generalist species.

Gut microbiota of Tephritidae have received great attention among those of phytophagous insects due to their promises for innovative pest management strategies (Deutscher et al., 2018; Noman et al., 2020), and because Tephritidae, which have a worldwide distribution, include some of the most economically damaging fruit and vegetable crop pests (Qin et al., 2015). The functional role of some particular bacterial taxa has been investigated within Tephritidae revealing links with nutritional provisioning (Behar et al., 2005), resistance to pathogenic bacteria (Behar, Yuval, et al., 2008), social interactions (Hadapad et al., 2016), pesticide resistance (Cheng et al., 2017), and foraging behavior (MacCollom et al., 2009). More recently, metabarcoding studies using next‐generation sequencing have helped describe the diversity and structure of the gut bacterial communities associated with wild Tephritid flies (Noman et al., 2020). These studies have uncovered a substantial diversity of gut bacteria with a strong predominance of the Proteobacteria phylum, including many genera of the Enterobacteriaceae family. Some conclusions, such as the lower diversity of microbial communities harbored in laboratory‐reared insects compared with field‐collected ones (Liu et al., 2016; Ras et al., 2017), and a core microbiota found only at the family level (De Cock et al., 2020; Deutscher et al., 2019), were shared by most studies. However, these studies also came to contrasted conclusions about the relative importance of host plants (Behar, Jurkevitch, et al., 2008; Majunder et al., 2019; Malacrinò et al., 2018; Ventura et al., 2018) or fruit fly species (De Cock et al., 2020; Morrow et al., 2015) in determining the composition and variation of gut bacterial communities in natural populations.

Here, we aimed at using the fruit fly community of Reunion Island to test the contributions of fly phylogeny, fly specialization, and fly sampling environment on the composition and structure of their bacterial gut microbiota. To do so, for each species, whose precise host range and phylogenetic history are known, we studied bacterial gut communities in samples from two contrasted environments (field vs. laboratory). Assessing the amount of network variation driven by different environmental and biological factors is still an experimental and statistical challenge (Joffard et al., 2019). Here, bacterial inventories were conducted using 16S metabarcoding with the Oxford Nanopore Technology, reported to confer a greater taxonomic resolution than Illumina at the genus level (Matsuo et al., 2021; Nygaard et al., 2020), and hence a key feature to dig into the diversity of Enterobacteriaceae. Moreover, meaningful network analyses relied on the framework recently proposed by Massol et al. (2021), based on two methods: (i) group decomposition followed by canonical correspondence analysis (CCA) and (ii) singular value decomposition (SVD) followed by redundancy analysis (RDA).

## 2 MATERIALS AND METHODS

### 2.1 Sample collection and DNA extraction

Details on each sample are provided in Table S1, Appendix S2. Field samples were collected in several localities between April and June 2018. When possible, flies were caught with pheromone traps in places where several host plants coexist. For species with no efficient trap, flies were collected from sets of infested fruits from a given locality (details in Table S1, Appendix S2). As pheromone traps only attract males, only male individuals were included in the study. Differences in gut composition between the sexes have been found nonsignificant in a preliminary study on *C*. *capitata* and *B*. *dorsalis* (not shown), and in previous studies on *B*. *dorsalis* (Andongma et al., 2015; Liu et al., 2018) and on another Tephritid species (*Bactrocera carambolae*, Yong et al., 2017). Laboratory flies were collected using mouth aspirator in populations maintained in the laboratory of Plant Populations and Bio‐agressors in Tropical Ecosystems Joint Research Unit (Saint‐Pierre, Reunion Island). All flies were stored for at least 48 h in fresh 90% ethanol at −30°C in a 10× liquid/fly volume ratio to optimize washing and dilution of any external bacteria. One hour prior to dissection, flies were rinsed at ambient temperature by successive buffers providing three more washes (75% ethanol, 50% ethanol, and 25% ethanol, 5 min each), while ensuring a progressive rehydration of the abdominal tissues for dissection. Dissection of the abdominal gut portion was performed on a sterilized glass slide with a pair of sterile tweezers under a stereomicroscope. The abdominal gut portion includes the midgut and the ileum of the hindgut, excluding anterior thoracic crop, foregut, and posterior rectum. For each sample, guts from around 30 males were dissected under sterile conditions and pooled.

DNA extraction from dissected guts was performed using the DNeasy Blood & Tissue Kit (Qiagen) following the manufacturer's instructions, adding 0.5% N‐lauroyl sarkosyl (Merck KGaA) for 30’, 65°C at the end of the lysis step. DNA was subsequently checked for quantity and quality with a NanoDrop 2000 (Thermo Fisher Scientific Inc.).

### 2.2 Gene amplification and MinION sequencing

For each sample, ~10 ng of DNA was amplified using specific primers that target the whole 16S rRNA gene (27F 5′‐AGAGTTTGGATCMTGGCTCAG‐3′; 1492R 5′‐GGTTACCTTGTTACGACTT‐3′), as well as subsequent specific barcodes using a 16S Barcoding Kit (SQK‐RAB204; Oxford Nanopore Technologies). After bead purification for removal of excess primers, amplification products were attached to rapid sequencing adapters before being loaded on a MinION flow cell for real‐time sequencing. Samples were analyzed in three separate experiments (RUN1, RUN2C, and RUN3 in barcodes cited in Table S1), each containing a mock community sample (more details in Appendix S1).

### 2.3 Bioinformatics

Basecalling, demultiplexing, and chimera removal were performed using Guppy v4.0.11 (https://community.nanoporetech.com). Reads were trimmed (only nucleotides between positions 60 and 1460 bp of the 16S rRNA gene were kept) and filtered (only sequences longer than 900 bp and above quality score Q10 were kept) using Nanofilt (De Coster et al., 2018), leading to a total of 268,960 sequences (ranging from 4693 to 36,902 across the 16 samples). Taxonomy was assigned by confronting reads to the Silva 138 database (Quast et al., 2013; Yilmaz et al., 2014) using VSEARCH 2020.8.0 (Rognes et al., 2016) embedded in QIIME 2 2020.8 (Bolyen et al., 2019), with a percentage of identity of 90%. A phyloseq object was produced and imported in R (McMurdie & Holmes, 2013; R Core Team, 2020). Examining mock samples revealed correct identification of mock taxa at all taxonomic levels, with relative abundances both very constant across runs and very close to the expectation (Figure S1 in Appendix S1). Among all reads, the percentage of successful assignment (proportion of total reads assigned to a taxon identified in the reference database) was 78.1% at phylum, class, order, and family levels. It dropped to 74.9% at the genus level and 34.0% at the species level. For further analyses, features were merged at the genus level, constituting an incidence table of 105 genera in 16 fruit fly samples. As the maximal relative abundance of a false‐positive taxon was 0.001 in mock community samples, the incidence table was filtered of taxa below this threshold relative abundance. This led to a final incidence table of 46 genera (list provided in Table S2) in 16 fruit fly samples (Table S3), used for all following statistical analyses.

### 2.4 Community diversity analyses

Community diversity was described as “effective numbers” (Hill, 1973; Jost, 2006, 2007) of bacterial genera within and among sample groups. The total (gamma) diversity of each group was multiplicatively partitioned into two components: (i) alpha diversity, the within‐group component; and (ii) beta diversity, the among‐group component, that is, the effective number of completely distinct communities present (Jost, 2006). Diversity decomposition was performed using inext (Hsieh et al., 2016) and the multipart() function of package vegan in R (Oksanen et al., 2020). To approximate uncertainty around diversity estimates, hierarchical bootstrapping was used. Further exploration of the variability of gut microbiota was conducted by nonmetric multidimensional scaling (NMDS) applied to the Bray–Curtis dissimilarity scores (Bray & Curtis, 1957).

### 2.5 Network analyses

To determine to what extent gut community structure is driven by fruit fly phylogeny, specialization, or sampling environment, we applied two network analysis methods exposed in Massol et al. (2021). To account for fly phylogeny, samples were divided into four groups based on fly genus: *Neoceratitis*, *Ceratitis*, *Dacus*, and the group formed by *Bactrocera* and *Zeugodacus*. The two latter genera are considered very close, to the point that until recently *Z*. *cucurbitae* was called *Bactrocera cucurbitae* (Virgilio et al., 2015; Zhang et al., 2010). Specialization groups are based on known host ranges in Reunion as inferred from long‐term observational data (Moquet et al., 2021) and divide samples into three groups: generalists (*Bactrocera* and *Ceratitis* species), specialists of Cucurbitaceae (*Z*. *cucurbitae* and *Dacus* species), and specialist of Solanaceae (*N*. *cyanescens*). Sampling environment opposes laboratory versus field samples.

The first method is based on inferring groups within the observed network. It compares this grouping of nodes (here samples) with groups based on factors at stake (here fly phylogeny, fly specialization, and fly sampling environment). The second method assesses the link between multivariate explanatory variables and network structure using redundancy analyses after SVD of the incidence matrix. In both methods, the significance of effects can be gauged through randomization.

Read counts can be poor proxies of abundances due to distortions inherited from the PCR process itself, and to representation biases of bacteria in reference databases (Brooks et al., 2016; Pollock et al., 2018). Therefore, it is generally considered safer to use presence–absence data. Here mock samples suggested both repeatable and moderate biases in abundance estimates from read counts. Thus we systematically conducted all community analyses on two versions of the sample × bacterial taxa incidence matrix: the weighted matrix, containing raw read counts; and presence–absence matrices, obtained by applying a threshold after rarefaction of the weighted matrix. While presence–absence matrices are generally considered to enable coping with uncertainty on relative abundance inference, they give rare taxa more weight into the analysis, as compared to weighted matrices. Importantly, because rarefaction is a random process, all analyses were applied on a distribution of presence–absence matrices, a safety step rarely done in microbiome studies.

#### 2.5.1 Binary incidence matrices

Presence–absence matrices can be obtained from read count data by setting a read count threshold below which a taxon is considered absent. Such threshold will only be meaningful if samples are first rarefied to a common total read count. However, rarefaction is a random process generating different matrices each time it is applied (examples are provided in Figure S2, Appendix S2). To account for this variability, we conducted community analyses on 1000 binary matrices. Each binary matrix was obtained by rarefying the read count matrix to 3000 reads (the smallest read count was 3250 for a *C*. *capitata* sample) and applying a threshold of three reads. This threshold value was determined by rarefying the mock samples to 3000 reads as well, and observing that false positives were never above three reads. For each observed binary matrix, a search of groups was conducted by maximizing network modularity with the leading eigenvector algorithm (Newman, 2006) using the R package igraph (Csardi & Nepusz, 2006). The membership of each fruit fly sample to inferred groups was summed up into a binary adjacency matrix (16 samples × 16 samples) with zero if samples belonged to two different groups and one if they belonged to the same group. The probability that two samples belong to the same group was then obtained as the proportion of the 1000 binary matrices leading to group these samples together.

#### 2.5.2 Null models for binary bipartite networks

Subsequent analyses required producing null distributions of network statistics. Following Massol et al. (2021), we produced a null model, called the configuration model, using the “curveball” algorithm (Strona et al., 2014), with functions “simulate” and “nullmodel” of R package vegan. In theory, each observed binary matrix is associated with a specific null distribution, which can only be approached by simulating multiple networks. For the sake of computation time, in the following, each “curveball”‐based test was performed using 1000 simulated networks for each of 100 observed binary matrices.

#### 2.5.3 Sample classification‐based tests

We assessed the effect of sampling environment, fly phylogeny, and fly specialization on gut bacterial community structure. We first proceeded one factor at a time and tested the congruence of sample classifications obtained through community‐search algorithms with those associated with external categorical variables, using the Normalized Mutual Information Index (NMI) (Astegiano et al., 2017) available through the function “compare” in the R package igraph (Csardi & Nepusz, 2006). The NMI takes values between zero, indicating no congruence, and one, corresponding to perfect congruence. One NMI value can be obtained for each rarefied matrix, and its associated significance can be inferred from 1000 corresponding matrices simulated under the null model as explained above. Here, mean NMI values were obtained on 1000 rarefied matrices, and the mean associated *p*‐value was obtained by comparing 100 rarefied matrices with 1000 corresponding simulations each.

To extend the same logic to multiple factors, we used CCA (ter Braak, 1986) using the function “cca” in the R package vegan. CCA allowed decomposing the variation of the community‐based classification of samples relatively to fly phylogeny, fly specialization and sampling environment. CCA can classically test the significance of a given “fraction” (e.g., chi‐square explained by factors *X* or *Y* once the effect of *Z* has been removed) by comparing the obtained *F*‐statistic with those yielded by randomizations of data rows (Peres‐Neto et al., 2006). Using the null model matrices, we could further test whether an effect that is deemed significant based on classical row permutations is purely due to heterogeneity in node degrees between communities (i.e., not significantly different from edge‐permuted expectation; richness effect) or not (affinity effect). Again, the whole CCA was conducted on 100 rarefied matrices, using 1000 corresponding simulations each.

#### 2.5.4 Singular value decomposition‐based tests

As a complementary approach, we also modeled the effects of fly phylogeny, fly specialization, and sampling environment on network structure using SVD coupled with RDA as explained in Massol et al. (2021). Any given *n* × *p* bipartite network can be approximated as two matrices (**L** and **R**) with a low number of columns and as many rows as nodes (*n* in **L**, *p* in **R**). Matrices **L** and **R** can be analyzed through a RDA to gauge how much variation among rows is explained by external variables. The number of vectors to keep after SVD was fixed after examining the congruence between communities inferred from SVD‐approximated networks with those inferred from the original network. SVD‐approximated networks were obtained by multiplying matrices **L** and **R** and setting a threshold for interaction prediction. Congruence between communities was obtained using the NMI between module partitions on a number of rarefied matrices.

#### 2.5.5 Weighted incidence matrix

A similar approach was applied to the weighted (read counts) incidence matrix, with the following differences. First, with weighted matrices, it is recommended to proceed through latent block models (LBMs) rather than modularity maximization to look for groups of nodes in networks (Leger et al., 2015). We therefore inferred groups using LBM with the R package sbm (Chiquet et al., 2021). We used a Gaussian distribution to model log‐transformed read counts. The best grouping was selected based on ICL criterion (Integrated Complete‐data Likelihood, a penalized likelihood criterion suited for clustering; Biernacki et al., 2000). Second, as no rarefaction step was used, analyses were conducted only once. Third, the null model comprised 10,000 matrices produced by Gaussian sampling on the outer product of margins of the log‐transformed weighted incidence matrix.

## 3 RESULTS

### 3.1 Descriptive analyses

**FIGURE 1:**
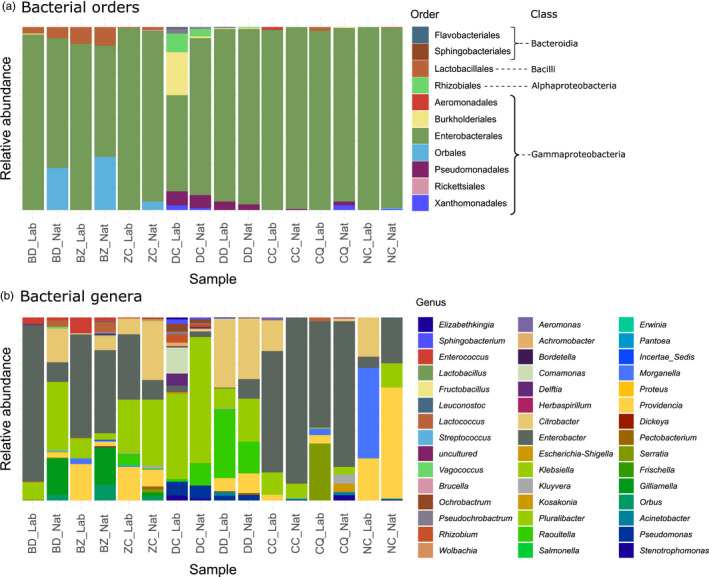
Relative abundances of bacterial taxa in the 16 samples. Within bars, colored areas indicate the proportion of each bacterial taxon in each sample from the weighted incidence matrix (Table S3), and white lines between two colored areas separate different genera. (a) Colors correspond to bacterial orders. (b) Colors indicate bacterial genera. Sample names are composed by the initials of fly species names (BD, *Bactrocera dorsalis*; BZ, *Bactrocera zonata*; CC, *Ceratitis capitata*; CQ, *Ceratitis quilicii*; DC, *Dacus ciliatus*; DD, *Dacus demmerezi*; NC, *Neoceratitis cyanescens*; and ZC, *Zeugodacus cucurbitae*) and the mention of sampling environment (Lab, laboratory; Nat, field, full details in Table S1)

The full bacterial composition of samples is provided in Figure 1 and Table S3 (Appendix S2). Rarefaction curves for each sample are provided in Figure S3 (Appendix S2). The 46 bacterial genera identified in the global dataset belonged to three phyla: Proteobacteria (97.7%), including two classes, eight orders, 15 families, and 36 genera; Firmicutes (2.0%), all of class Bacilli and order Lactobacillales, with eight genera in six families; and Bacteroidota (0.3%), only represented by two genera (Table S2). The genera above 1% in total abundance belonged to two classes (Bacilli and Gamma‐Proteobacteria), with an overrepresentation of the latter (97.1%). Among the 11 detected orders, four were above 1% in total abundance (Enterobacterales, Lactobacillales, Orbales, and Pseudomonadales), with an overrepresentation of Enterobacterales in all samples (52.5%–99.9%). Only five families (Enterobacteriaceae, Enterococcaceae, Morganellaceae, Orbaceae, and Pseudomonadaceae) were above 1% in total abundance, with an overrepresentation of Enterobacteriaceae (69.1%) in all fly species but *N*. *cyanescens*, dominated by the phylogenetically close Morganellaceae. Only nine genera were above the 1% threshold (*Enterobacter*, *Klebsiella*, *Citrobacter*, *Providencia*, *Morganella*, *Raoultella*, *Gilliamella*, *Pseudomonas*, and *Enterococcus*; Table S3). Some bacterial taxa have preferential associations with fly phylogenetic groups. The Bacteroidota phylum and the Alpha‐Proteobacteria class tended to associate with samples of *Dacus*. The Firmicutes phylum associated with *Bactrocera* and *Zeugodacus* samples. Some bacterial taxa had variable prevalence across sampling environments as well. Examples of genera with variable prevalence between laboratory and field samples include *Enterobacter*, *Morganella*, and *Citrobacter*. Finally, for some bacteria, the prevalence seemed determined by both fly phylogeny and sampling environment, such as the Orbales class, mainly found in field samples of *Bactrocera* and *Zeugodacus*.

### 3.2 Diversity partitioning

The total (gamma) diversity of the 16 samples was 8.40 (95% CI 5.72–10.18) genus equivalents.

**FIGURE 2:**
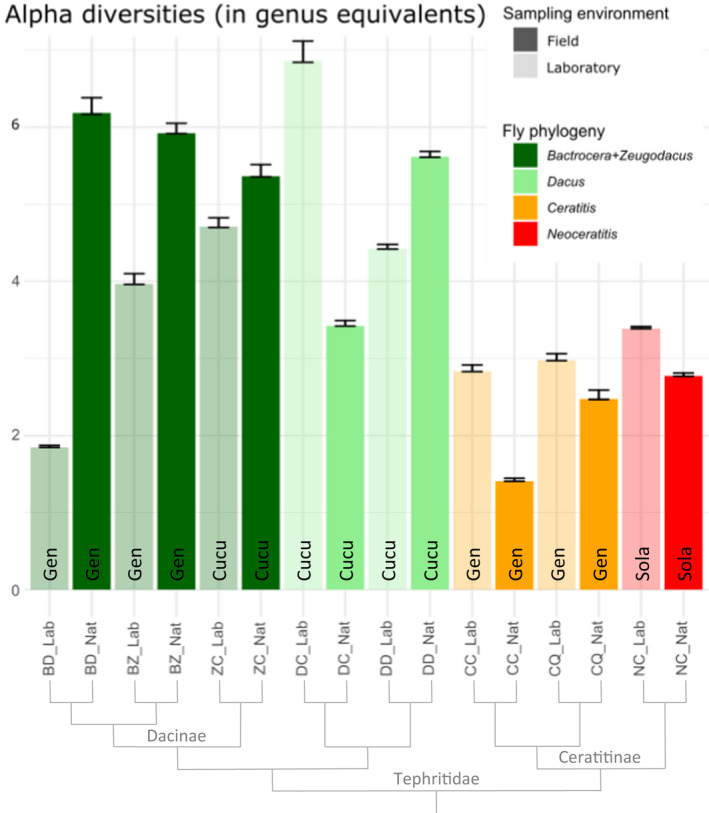
Alpha diversity for each sample. Bars indicate Hill numbers of order one (exponential of Shannon diversity, in genus equivalents). Bar colors correspond to fly phylogenetic groups, and shading stands for sampling environment. Codes at the bottom of each bar designate fly specialization groups: “Gen” for generalist species, “Cucu” for specialists of Cucurbitaceae, and “Sola” for the specialist of Solanaceae. Error bars represent 95% confidence intervals estimated by bootstrapping

The alpha diversity of samples ranged from 1.41 (95% CI 1.41–1.45, for field *C*. *capitata*) to 6.86 (95% CI 6.84–7.11, laboratory *D*. *ciliatus*) genus equivalents, with a mean of 4.01 (SE 0.41) (Figure 2). Average alpha diversity of laboratory (3.87, SE 0.54) and field samples (4.14, SE 0.65) was close. For all Dacinae samples but *D. ciliatus*, the field sample was more diverse than the laboratory sample, whereas in Ceratitinae, the laboratory sample was more diverse than the field sample (Figure 2). Among laboratory samples, there was no clear link between alpha diversity and the number of generations spent by populations in the laboratory prior to sampling (Appendix S2, Figure S4). Alpha diversity did not seem to particularly correlate with specialization (Figure 2): Diversity was not greater in generalists (3.45, SE 0.63) than in specialists of Cucurbitaceae and Solanaceae (5.06, SE 0.48 and 3.08, SE 0.31, respectively). In contrast, sample diversity tended to differ between phylogenetic groups (Figure 2). Dacinae samples had an average of 4.83 (SE 0.47) genus equivalents, while Ceratitinae samples had only 2.64 (SE 0.27).

Pairwise beta diversity between samples ranged between 1.03 (between laboratory *B*. *dorsalis* and field *C*. *capitata*) and 1.91 (between laboratory *N*. *cyanescens* and *D*. *ciliatus*). Differentiation among bacterial communities was not particularly structured by sampling environment, as beta diversity between laboratory and field samples was 1.12. In contrast, beta diversity, even though estimated on the whole dataset (i.e., with both laboratory and field samples), tended to be higher between specialization groups (1.70) and between host phylogenetic groups (1.83).

**FIGURE 3:**
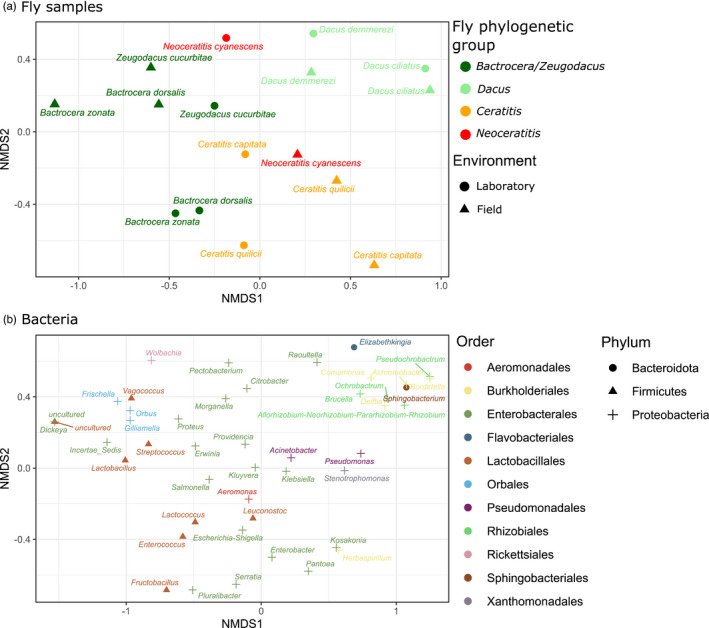
Nonmetric multidimensional scaling (NMDS) ordination of fruit fly gut microbiome communities compared using the Bray–Curtis similarity coefficient of bacterial genera relative abundances. (a) Sample ordination. Colors represent fly phylogeny. Symbols represent sampling environment. (b) Ordination of bacterial genera. Colors represent bacterial orders. Symbols correspond to phyla

Nonmetric multidimensional scaling attained a stress value of 0.1932. It tended to group samples by phylogenetic group, rather than by sampling environment or fly specialization (Figure 3), a result also observed in NMDS ordination of presence–absence matrices (Figure S5 in Appendix S2). *Dacus* samples seemed to distinguish from other samples by higher relative abundance of Bacteroidota (genera *Elizabethkingia* and *Sphingobacterium*), lower relative abundance of Firmicutes (eight genera, all of class Bacilli, order Lactobacillales), and higher relative abundance of several genera from two orders of Alpha‐Proteobacteria (Rhizobiales and Burkholderiales). Field *Bactrocera* and *Zeugodacus* samples tended to preferentially associate with the Lactobacillales *Streptococcus*, *Lactobacillus*, and *Vagococcus*, and among Gamma‐Proteobacteria, with three Orbales genera (*Frischella*, *Gilliamella*, and *Orbus*) and some Enterobacterales genera. Field Ceratitinae mainly differed from others by their association with Enterobacterales genera such as *Kosakonia* and *Pantoea* and with the Burkholderiales genus *Herbaspirillum*.

### 3.3 Network analysis

#### 3.3.1 Sample groups

**FIGURE 4:**
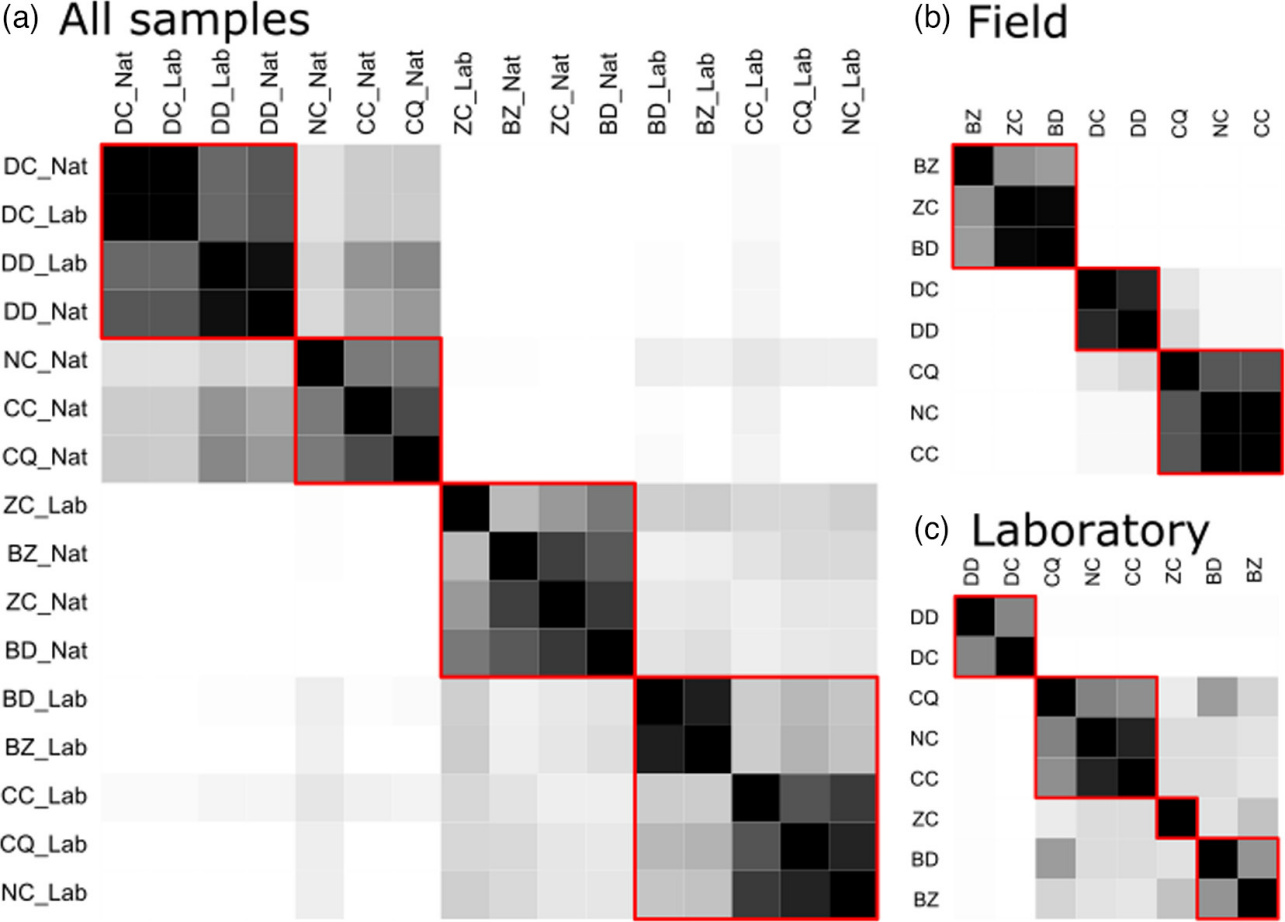
Mean clustering of samples based on gut microbial groups identified from 1000 rarefied presence–absence matrices. Color gradient corresponds to the percentage of rarefied matrices in which two samples are found in the same cluster (white = 0%, black = 100%). Red contours indicate the most common clustering. (a) All 16 samples. (b) Only field samples. (c) Only laboratory samples

Applying the leading eigenvector community‐search algorithm to the 16 samples over 1000 observed presence–absence matrices led to identify 4.203 groups of nodes in the network on average (SE = 0.057, Figure 4a and Figure S6 in Appendix S2), with a relatively high and significant modularity score (*Q* = 0.301, SE = 0.0005, 95% PI = 0.262–0.328, left panel of Figure S7, Appendix S2). Over a random subset of 100 observed binary matrices, the *p*‐value of the observed modularity had a mean of 0.005 (SE = 0.002, 95% PI = 0.000–0.0461, right panel of Figure S7, Appendix S2), suggesting that observed matrices were more structured than expected under the null model. All binary matrices separated at least two relatively stable groups (Figure 4a). The first group tended to split into two subgroups: (i) all *Dacus* samples, whatever their environment, most frequently grouped together (72%–95% of observed binary matrices), and (ii) field samples of Ceratininae species (genera *Ceratitis* and *Neoceratitis*). Field *Ceratitis* species were associated with 80% of observed binary matrices. *Neoceratitis* was less frequently associated with them (68% of observed binary matrices). Samples of both subgroups (*Dacus* and field Ceratitinae) were associated with 22.4%–63.6% of observed binary matrices. The second group was also composed of two main subgroups, with more variable composition: (i) *Zeugodacus* samples and field *Bactrocera* samples (percentages varying from 51% to 84%), and (ii) all remaining samples, that is, laboratory *Bactrocera* and Ceratitinae samples.

The same community‐search algorithm also revealed an average of 3.45 (SE = 0.02) groups among field samples only and 3.81 (SE = 0.03) groups among laboratory samples, with congruent compositions with the 16‐sample grouping (Figure 4b,c).

**FIGURE 5:**
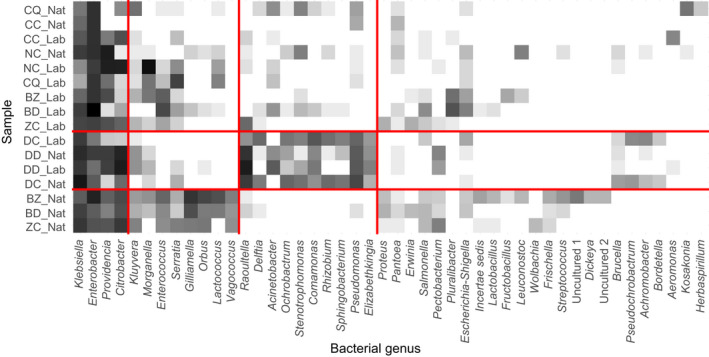
Groups identified from the full read count matrix. Log‐transformed read counts are represented on a continuous gradient from white for log10(reads + 1) = 0, to black for log10(reads + 1) = 4.4. Red lines delimit clusters identified under the best latent block model

On the whole weighted incidence matrix, LBM identified three groups of samples (Table S4, Figure S8 in Appendix S2, and Figure 5): one with field samples of *Zeugodacus* and *Bactrocera* species, one with all *Dacus* samples, and the remaining samples (all Ceratitinae samples and laboratory samples of *Zeugodacus* and *Bactrocera*). On field samples only, two groups of samples were found: one with Dacinae species (*Bactrocera*, *Dacus*, and *Zeugodacus*) and one with all Ceratitinae species (*Ceratitis*, and *Neoceratitis*) (Figure S9, Appendix S2). On laboratory samples, no group was identified (Figure S9, Appendix S2).

#### 3.3.2 Congruence of classifications

**FIGURE 6:**
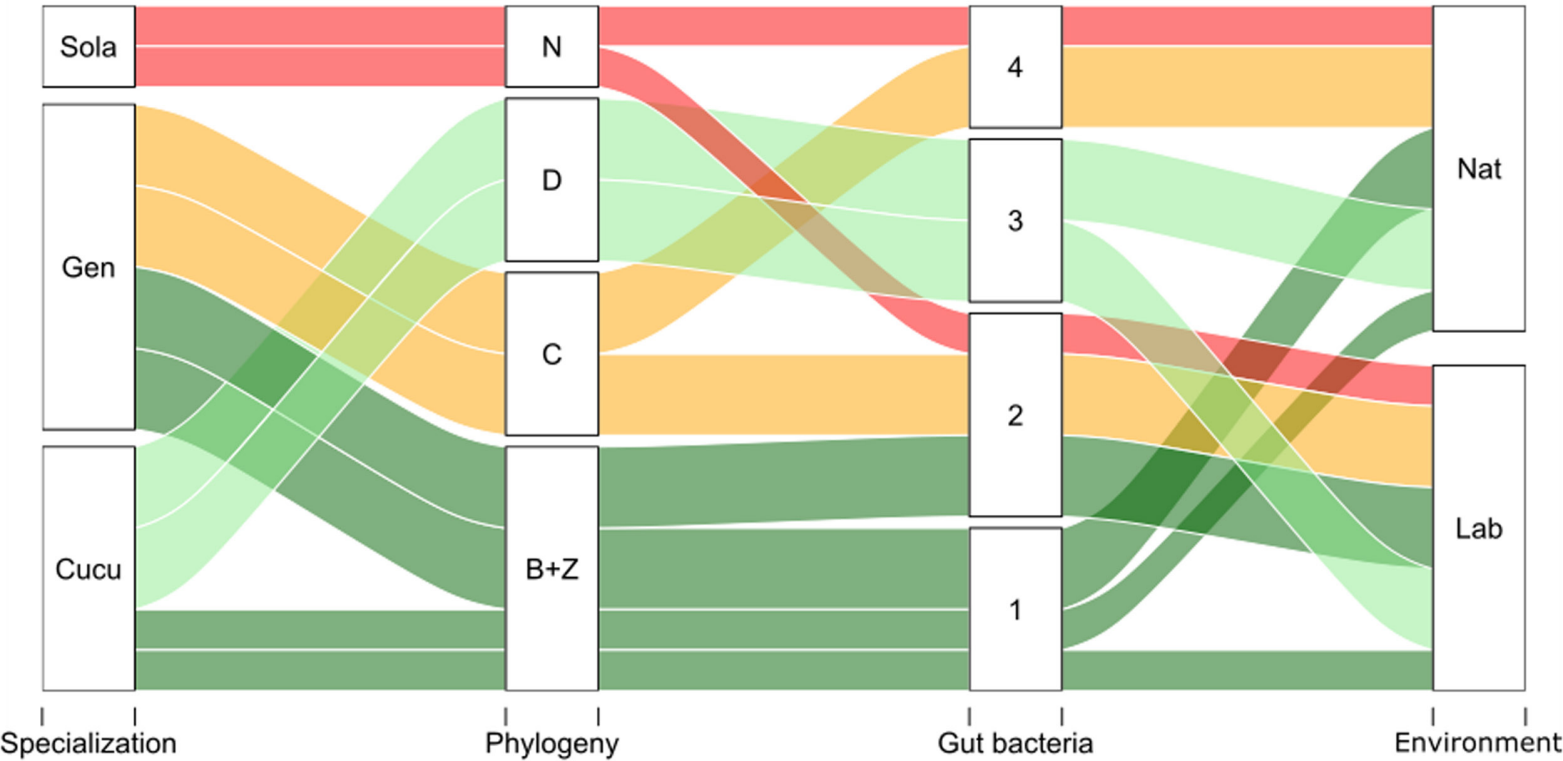
Alluvial plot showing how the 16 samples group according to fly specialization, fly phylogeny, fly sampling environment, and gut bacterial composition. Fly specialization counts three groups of samples: the specialists of Solanaceae (the two *Neoceratitis cyanescens* samples), the generalists (the eight *Bactrocera* and *Ceratitis* samples), and specialists of Cucurbitaceae (*Dacus* and *Zeugodacus* samples). The factor “Phylogeny” groups samples by fly genus: N, D, C, and B+Z stand for *Neoceratitis*, *Dacus*, *Ceratitis*, and *Bactrocera* or *Zeugodacus*, respectively. Environment separates laboratory samples (Lab) from field samples (Nat). The last grouping factor (Gut bacteria) is the mean clustering of samples based on gut microbial subcommunities identified from 1000 rarefied presence–absence matrices (the four red squares in Figure 4a). Colors correspond to fly phylogeny

Distributions of the congruence indices (NMI) are provided in Table 1. The communities found in the whole network were most congruent with genus‐level fly phylogeny (mean *p*‐value < 0.05). Other classifications of samples, that is, based on higher‐level fly phylogeny (Ceratitinae vs. Dacinae), sampling environment, or fly specialization, were not statistically more congruent with gut microbiota‐based clustering than expected by chance (see Figure 6 for an illustration).

**TABLE 1:** Congruence between classifications of samples based on gut bacteria presence–absence data, and potential determinants of community structure

Classification of samples		NMI with clustering based on gut bacteria			*p*‐Value	
Factor	Modalities	Mean	SE	Percentile interval	Mean	SE
Phylogeny	Dacinae–Ceratitinae	0.169	0.002	0.055–0.302	0.462	0.030
	*Ceratitis–Neoceratitis*–(*Bactrocera* + *Zeugodacus*)–*Dacus*	0.572	0.003	0.427–0.746	0.044	0.007
Sampling environment	Field–laboratory	0.267	0.004	0.056–0.497	0.210	0.026
Specialization	Generalists—specialists of Cucurbitaceae—specialist of Solanaceae	0.304	0.003	0.111–0.466	0.372	0.029

Note

Congruence was estimated by NMI obtained from 1000 observed rarefied matrices. NMI ranges from zero for no congruence, to one for perfect congruence. Significance of these NMI values was obtained from 100 comparisons between one observed rarefied matrix and 1000 random null model matrices.

Abbreviation: NMI, Normalized Mutual Information Index.

#### 3.3.3 Canonical correspondence analyses

The results of CCA applied to the communities found on both presence–absence and read count data confirmed the results found by congruence comparisons. Fly phylogeny significantly explained gut bacterial communities, irrespective of the removal of the effects of environment, host specialization, or both (Table 2A,B). On presence–absence data, none of the models omitting phylogeny or removing the effect of phylogeny was significant (Table 2A). Significant models were all doubly significant (with permutation tests on rows and edges), indicating both node richness and affinity differences between groups. On read count data, the models associated with the lowest *p*‐values included both phylogeny and sampling environment (Table 2B). Most significant effects were not significant under edge permutations, indicating an effect mainly driven by differences in gut microbiota richness between groups of nodes.

**TABLE 2:** Canonical correspondence analyses (CCA) between groups based on gut microbiota and sampling environment (samp env), fly specialization (fly spe), and fly phylogeny (fly phy)

Formulas	(A) Presence–absence data					(B) Read counts				
	*p*‐Value (row perm.)		*p*‐Value (edge perm.)			Chi^2^	*F*	*p*‐Value (row perm.)	*p*‐Value (edge perm.)	
	Mean	SD	Mean	SD						
samp env	0.163	0.204	—	—	NS	0.250	2.000	0.269	—	NS
samp env + Cond (fly spe)	0.118	0.163	—	—	NS	0.250	2.678	0.119	—	NS
samp env + Cond (fly phylo)	0.071	0.127	—	—	NS	0.250	7.200	0.044	0.027	**
samp env + Cond (fly spe) + Cond (fly phy)	0.070	0.127	—	—	NS	0.250	6.600	0.041	0.031	**
fly spe	0.255	0.218	—	—	NS	0.630	3.216	0.049	0.389	*
fly spe + Cond (samp env)	0.202	0.186	—	—	NS	0.630	3.653	0.023	0.395	*
fly spe + Cond (fly phy)	0.410	0.241	—	—	NS	0.000	0.000	NA	—	NS
Cond (samp env) + fly spe + Cond (fly phy)	0.334	0.252	—	—	NS	0.000	0.000	NA	—	NS
fly phy	0.018	0.031	0.020	0.042	**	1.333	10.000	0.000	0.283	*
fly phy + Cond (samp env)	0.010	0.026	0.014	0.035	**	1.333	14.933	0.000	0.113	*
fly phy + Cond (fly spe)	0.024	0.033	0.031	0.057	**	0.704	6.861	0.007	0.486	*
Cond (samp env) + Cond (fly spe) + fly phy	0.015	0.027	0.021	0.048	**	0.704	10.133	0.001	0.248	*
samp env + fly spe	0.096	0.127	—	—	NS	0.880	3.664	0.017	0.211	*
samp env + fly phy	0.008	0.022	0.010	0.032	**	1.583	14.250	0.000	0.067	*
fly spe + fly phy	0.035	0.055	0.036	0.068	**	1.333	7.500	0.002	0.471	*
samp env + fly spe + fly phy	0.014	0.037	0.016	0.049	**	1.583	11.400	0.000	0.244	*

Note

Significance of individual fractions was tested by row permutations or edge permutations. Significance based on row permutations is evaluated based on the corresponding *p*‐value, and estimated as the probability that a randomized version of the explained contingency table, once removed the effect of conditioning variables, obtains a *F*‐statistic equal or larger to the one obtained with real data. Significance based on row permutations is indicated with a star. NS stands for not significant. Significance based on edge permutations is only computed for effects significant with the first test. It is obtained as the probability that a randomized version of the contingency table, keeping node degrees constant, obtains a *F*‐statistic equal or larger to the one obtained with real data. Double significance is indicated with two stars. (A) Mean‐*p*‐values associated with any given combination of factors (with SD) obtained from 100 rarefied presence–absence matrices, with 1000 simulated null matrices each. (B) Chi‐square, and *F* and *p*‐values associated with any given combination of factors obtained for the read count matrix.

#### 3.3.4 Singular value decomposition and redundancy analyses

**FIGURE 7:**
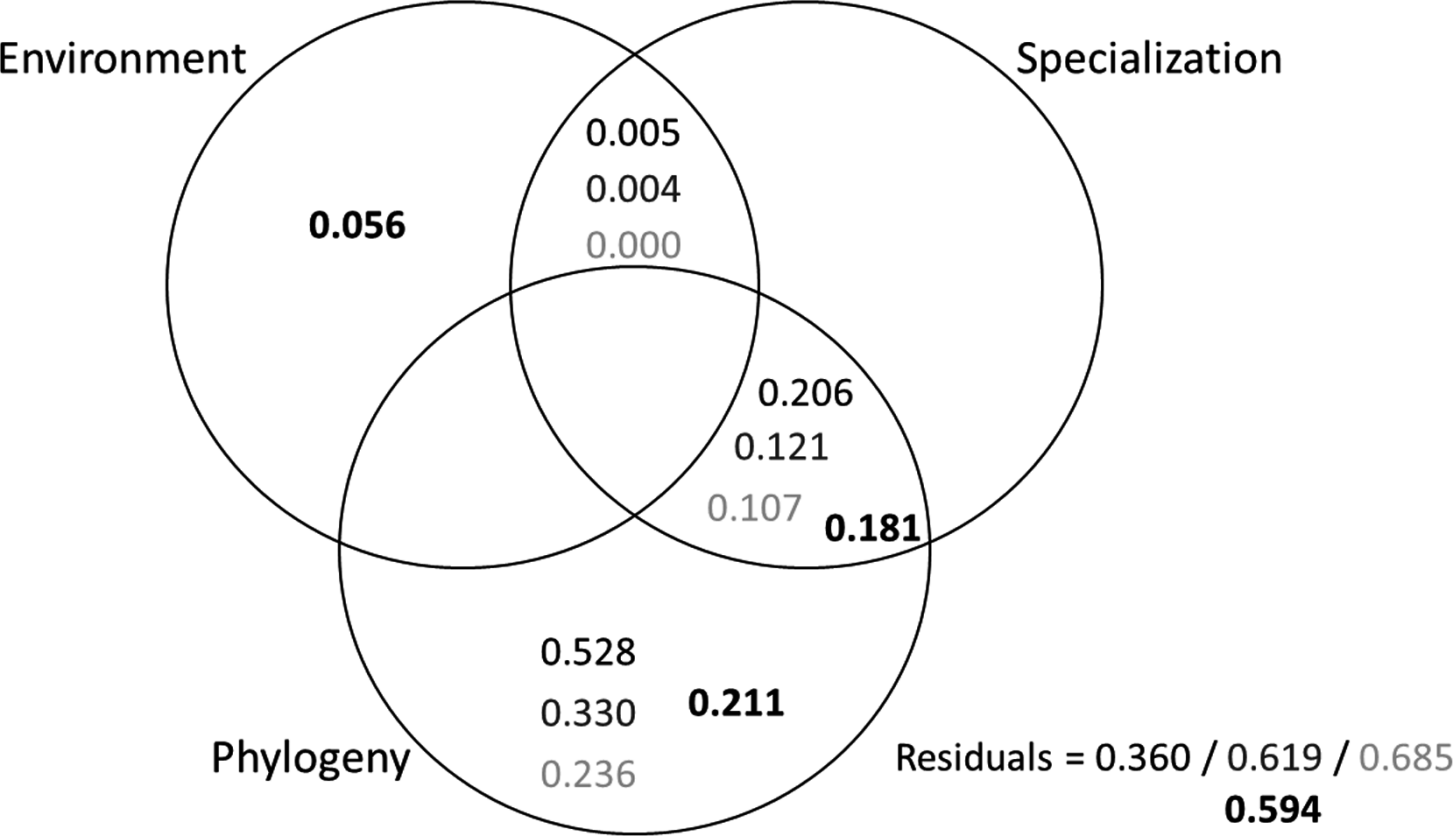
Venn diagrams representing the partition of variation (redundancy analysis) within the reduced matrices obtained by singular value decomposition of incidence matrices. Given values are non‐negative adjusted *R* ^2^ of individual fractions. Negative adjusted *R* ^2^ are omitted for clarity. Bold numbers were obtained on the read count matrix approximated with four retained vectors. Nonbold numbers were obtained from presence–absence matrices approximated with two (upper black numbers), three (middle dark gray numbers), and four (bottom light gray numbers) vectors. These latter values are means from 100 presence–absence matrices. All standard errors ranged between 0.002 and 0.004

As a first step, the number of vectors required to faithfully approximate incidence matrices was determined by estimating the congruence between groups obtained from the approximated matrices with groups obtained on the full matrix. On presence–absence data, congruence tended to increase with the number of vectors retained, but the first local maximum occurred between two and four vectors retained depending on the rarefied matrix. On read counts, a single local maximum was observed at four vectors. Adjusted *R* ^2^ values of individual fractions are given for these various options (Figure 7) and are all very congruent. Residual error (i.e., variance not explained by fly phylogeny, fly specialization of sampling environment) increased steadily with the number of vectors, but remained high (>36%). For any given number of vectors, fly phylogeny had the highest adjusted *R* ^2^, followed by the interaction between fly phylogeny and fly specialization. Sampling environment explained a marginal part of variance on read counts only.

## 4 DISCUSSION

The gut bacterial microbiota of eight Tephritidae species were described using Oxford Nanopore MinION full‐length 16S metabarcoding. At taxonomic levels ranging from phylum to family, the abundance of bacterial taxa was found congruent with former descriptions obtained with Illumina MiSeq data from other Tephritidae species (for a review, see Noman et al., 2020 and Raza et al., 2020), and from some of these species in other geographic area (De Cock et al., 2020; Hadapad et al., 2016; Malacrinò et al., 2018). Enterobacteriaceae, identified as the most prevalent family in nearly all samples, are reportedly transferred vertically in some species (Aharon et al., 2013; Lauzon et al., 2009; Majunder et al., 2019) and thus are considered important for Tephritid development and physiology. At genus level, existing published studies exhibit substantial variability in descriptions of abundant bacteria. Here, thanks to the higher resolution of long‐read metabarcoding, 46 genera were found, the most abundant of which have also been described in other Tephritidae studies, including *Enterobacter*, *Klebsiella*, *Citrobacter*, *Providencia*, *Morganella*, and *Raoultella* (for a review, see Noman et al., 2020 and Raza et al., 2020). In contrast, some genera mentioned as frequent in other Tephritid studies were only found at low abundances here, as for example, *Acetobacter*, *Escherichia*, *Pectobacterium*, and *Serratia*. Whether these discrepancies are due to methodological issues or natural variability cannot be fully deciphered here. In the present study, only one pooled sample by fly species and fly sampling environment was studied, hampering considerations on natural intraspecific variability in gut microbiome composition. For some abundant taxa, results of functional studies monitoring fruit fly fitness are worth mentioning. For instance, *Enterobacter* and *Klebsiella* enhance larval nutrition (Noman et al., 2020 and references herein). An addition of *Klebsiella* in controlled conditions increases pathogen resistance of *C. capitata* (Ben‐Ami et al., 2010). In the same way, Cheng et al. (2017) have described the resistance of *Citrobacter* to resist trichlorfon insecticide in *B*. *dorsalis*. Finally, *Enterobacter*, *Raoultella*, *Klebsiella*, *Citrobacter*, and *Providencia* may also play a role in sexual and host plant attractiveness (Raza et al., 2020 and references herein). In contrast, *Providencia* and *Morganella* have been described as potential pathogens of fruit flies (M'Saad Guerfali et al., 2018; Salas et al., 2017), thus able to decrease fruit fly fitness.

The recent accumulation of sequence data from microbial communities has made some authors plead for an extension of community analyses beyond the exploration of alpha‐ and beta‐diversity patterns in order to detect robust associations between microorganisms and hosts (Barberán et al., 2012; Burns et al., 2016). Here, classic diversity analyses were supplemented with network‐based clustering techniques (Massol et al., 2021) using either the leading eigenvector of presence–absence matrices (Csardi & Nepusz, 2006) or LBMs for the read count matrix (Chiquet et al., 2021). Such techniques may help cluster bacterial taxa according to their pattern of association with host flies and gut samples based on their microbial community profiles. Clustering methods may thus provide a natural way of revisiting the notion of core microbiome. Here, the use of various clustering analyses (on all, only laboratory or only field samples, and on read count vs. presence–absence data) supported at least three congruent groups of samples: all *Dacus* samples, field *Bactrocera* and *Zeugodacus* samples, and other samples (Ceratitinae and laboratory *Bactrocera* and *Zeugodacus*). Within this latter group, presence–absence matrices suggested possible subgrouping of Ceratitinae vs. the Dacinae *Bactrocera* and *Zeugodacus*. Clustering of bacteria highlighted a group of bacterial genera accounting for more than half of the bacterial prevalence in all samples: the Enterobacteriaceae *Citrobacter*, *Enterobacter*, and *Klebsiella*, and the Morganellaceae *Providencia*. This group, also supported by numerous studies of Tephritidae microbiota (Behar, Jurkevitch, et al., 2008; Hadapad et al., 2016; Liu et al., 2016; Morrow et al., 2015; Ventura et al., 2018; for a review, see Noman et al., 2020 and Raza et al., 2020), could be considered as a core microbiota at the scale of the Tephritidae family. A second group of bacterial genera, common in field *Bactrocera* and *Zeugodacus* samples, was rare in *Dacus* samples and of variable abundance in other samples. This group included Enterobacterales (*Kluyveria*, *Morganella*, *Serratia*), two Orbales (*Gilliamella* and *Orbus*), and all the Lactobacillales (represented by *Enterococcus*, *Lactococcus*, and *Vagococcus*). Associations between *Lactococcus* and *B. zonata* have already been described (De Cock et al., 2020). The third group of bacteria was preferentially associated with *Dacus* samples: the Alpha‐Proteobacteria genera *Rhizobium* and *Ochrobactrum*, the Bacteroidia genera *Elizabethkingia* and *Sphingobacterium*, and among Gamma‐Proteobacteria, genera belonging to diverse orders (the Burkholderiales *Comamonas* and *Delftia*, the Pseudomonadales *Acinetobacter* and *Pseudomonas*, the Xanthomonadales *Stenotrophomonas*, and the Enterobacterales *Raoultella*). Other bacteria fell in a fourth cluster, with no obvious association profile, likely due to their low abundances. These nonrandom associations of bacterial taxa with fly samples were further confirmed by NMDS. Interestingly, some preferential associations occurred at higher taxonomic scales. For instance, Bacteroidota and Alpha‐Proteobacteria were mainly associated with *Dacus* samples. In contrast, Firmicutes were completely absent from *Dacus* samples, as well as from field *Ceratitis* samples. Many preferential associations involved different families of Gamma‐Proteobacteria and different genera within the Enterobacteriaceae family, raising the need for a finer taxonomic resolution within this key bacterial family. Because of the genuine sympatry of the eight species, the highlighted clusters could not be considered as determined by geographic differentiation in microbial pools, and therefore offer candidate taxa for subsequent functional analyses.

The different methods used to evaluate the relative importance of fly phylogeny, fly specialization, and fly sampling environment converged to the conclusion that fly phylogeny was the main factor explaining microbial profile. In contrast, host ecology (i.e., fly specialization and sampling environment) did not imprint significantly gut microbial communities. For instance, samples of the species *Z*. *cucurbitae*, a specialist of Cucurbitaceae host plants, systematically grouped with the *Bactrocera* samples, which correspond to generalist species, and not with *Dacus* samples, which share the same host range but are more distant phylogenetically. Samples of both *Ceratitis* generalist species tended to group with the other Ceratitinae species, *N*. *cyanescens*, a specialist of Solanaceae, rather than with *Bactrocera* samples, which share this generalist niche. The methodological robustness of our results was achieved by the observation of both read count (which tend to give more weight to very abundant species) and of presence–absence (which are more affected by rare taxa) data. Our results, suggesting that microbial profiles are affected by host phylogeny rather than host ecology, are thus unsupportive of the hypothesis formulated by Zhao et al. (2016), according to which the Tephritidae gut community membership would be controlled by host genetics, while bacterial abundance would be driven by nongenetic factors.

Phylogenetic determinism of gut microbial communities has been observed in a diversity of taxonomic groups, including nematodes, numerous insect clades, fish, mammals, and hominids (Moran et al., 2019). Such a pattern may indicate a shared, faithful history between hosts and their microbes (Brooks et al., 2016). This process, sometimes referred to as “phylosymbiosis,” has been observed in *Nasonia* wasps, and is prone to co‐adaptations between hosts and microbes (Brucker & Bordenstein, 2012). Alternatively, this same pattern may very well be driven by physiological, morphological, ecological, or behavioral similarities in closely related hosts that lead to similar environmental filtering of microbial pools (Moran & Sloan, 2015). In Tephritidae species of Reunion Island, host ecology likely determines social interactions. *Bactrocera* and *Ceratitis* species on the one hand, and *Dacus* and *Zeugodacus* species on the other hand, are often found developing in the same fruits (Facon et al., 2021). We do not find such clustering when analyzing their gut microbiota, which suggests that such social interactions unlikely contribute to the structure of gut microbiota in the studied species.

The present results do not conform to the hypothesis that generalist species should have more diverse gut microbial communities (Deb et al., 2019), as confirmed in scavengers and omnivores (Shukla et al., 2016; Yadav et al., 2015; Yun et al., 2014). The generalist *Ceratitis* species had lowest gut microbial diversity (around two genus equivalents). Their relative specialist of Solanaceae, *N*. *cyanescens*, had slightly higher microbial diversity, noticeably due to a relatively high abundance of Morganellaceae. The specialists of Cucurbitaceae (*Dacus* and *Zeugodacus* species) had the highest microbial diversity (around five genus equivalents), whereas their relative generalists of genera *Bactrocera* had less diverse gut content (around four genus equivalents). The observation that fruit fly specialization does not significantly imprint gut microbial communities is rather a surprise (but see De Cock et al., 2020, for a first mention). Plants present numerous nutritional and defensive challenges to phytophagous insects. A growing body of research emphasizes the potential contribution of symbiotic microbes to phytophagous diets (Feldhaar, 2011; Felton & Tumlinson, 2008; Oliver et al., 2010). Nevertheless, the accumulated evidence is mixed and requires further sampling and functional analyses of the fruit fly gut microbiota. Gut microbiota respond more to host phylogeny rather than to host ecology in aphids (McLean et al., 2019) and in lycaenid butterflies (Whitaker et al., 2016). The reverse has been observed in both fruit‐feeding and mycophagous drosophilid species (Adair et al., 2020), ants (Anderson et al., 2012), and beetles (Blankenchip et al., 2018).

In interaction with phylogeny, the environment of sampling (here field vs. laboratory) had a detectable moderate effect on gut communities, in terms of both diversity and composition. In most Dacinae species (all but *D*. *ciliatus*, the most recent laboratory population), laboratory populations had less diverse gut microbiota as compared to natural populations. This observation has been made repeatedly in Tephritidae, such as *B*. *tryoni* (Morrow et al., 2015), *B*. *oleae* (Ras et al., 2017), and a range of arthropod species (Belda et al., 2011; Ng et al., 2018; Pérez‐Cobas et al., 2015; Staubach et al., 2013; Xiang et al., 2006). In clear contrast with these observations, we found that Ceratitinae laboratory populations were more diverse than field ones. Besides, except for *Dacus* samples, laboratory and field populations tended to differ strongly in terms of composition. In the present study, laboratory populations almost missed the class of Orbales (as already observed by Martinson et al., 2017), several Enterobacterales and Lactobacillales. Laboratory samples were also less dominated by the genus *Klebsiella*. Three genera were only present in the laboratory (*Aeromonas*, *Fructobacillus*, and *Pluralibacter*), and some genera very rare in nature had important relative abundance in the laboratory, such as the Yersiniaceae *Serratia* and the Morganellaceae *Morganella*. Some differences between laboratory and field populations contrasted with former observations, for example, describing a dominance of *Providencia* or *Acinetobacter* in laboratory populations (Ben‐Yosef et al., 2015; Kounatidis et al., 2009).

The laboratory populations are occasionally supplemented with field individuals so that this differentiation cannot be explained by pure drift. This suggests a genuine recomposition of gut microbiota in laboratory populations in response to local conditions (missing nutriments, antifungal treatment…). Interestingly, while laboratory populations did share very similar conditions, the constraint of phylogeny on microbial communities was still much apparent. Such a result has implications for fruit fly management strategies based on sterile insect techniques, as well as for ecological and evolutionary studies using laboratory populations. Many studies have mentioned a loss of competitiveness of laboratory flies vs. field individuals. It is possible that part of this lesser fitness is due to gut microbiota modifications, that it could be attenuated by working of microbiota restoration, and that the intensity of this effect is species‐specific.

Overall, gut microbiota were strongly imprinted by fly phylogeny, but could be subject to important restructuring in the face of new environmental conditions. As a consequence, the observed lack of correlation between gut microbiota and both fly specialization and fly sampling environment is a surprise and needs to be addressed. It is possible that most gut microbes have functions other than fruit digestion (Ben‐Ami et al., 2010; Cheng et al., 2017; Hadapad et al., 2016) or that there is functional redundancy; that is, microbial functions can be ensured by different taxa (Moya & Ferrer, 2016). Importantly only adults were studied here. In fruit flies, adults do not eat much, and only larvae feed on fruit. Yet, adult gut bacteria are the ones with a chance to be vertically transmitted. It could then be advantageous for flies that adults keep and transmit bacteria beneficial to larvae, including bacteria associated with plant use. Alternatively, it is possible that some useful gut bacteria are transitorily acquired by larvae in the fruit they grow in, before being eliminated at metamorphosis. Such ability to select and breed useful bacteria in the environment would confer an adaptive plastic response to host plants. These bacteria, which would likely differ across sampling environments, would not be detectable in studies focused on adults as here. And studying the contribution of gut microbiota to fly host range would require studying larvae as well. In Tephritidae, comparisons between larval and adult gut content are too rare and divergent for any conclusion to be drawn as to whether or not larvae acquire essential bacteria in the fruit, which would be released upon metamorphosis. Evidence from comparisons between larvae and fruits does not point toward this hypothesis. In *B*. *tryoni*, larval gut microbiota were more diverse than those of fruits and not influenced by fruit (Majunder et al., 2019). But in other flies, such as drosophilid flies, host ecology seems to have detectable impact on larval gut microbiota (Chandler et al., 2011). Another possible factor affecting gut microbiota composition and transmission might be the effect of larval diet on adult immunity (Fellous & Lazzaro, 2010). Adult immunity is likely the final gate filtering microbial taxa inherited by their progeny, and thus factors affecting immunity, including diet and other environmental conditions, could explain phylosymbiosis (or the lack thereof). Besides, the interactions between the host and a given microbe could be highly dependent on the other microbes constituting the microbiota. In such cases, a high rate of vertical transmission for a given microbe could greatly influence the rest of the microbiota. Dissecting the contribution of niche‐based processes in the assembly of the gut microbiota is therefore still an important challenge for future research using both field samples and gnotobiotic animals in controlled conditions.

## CONFLICT OF INTEREST

The authors declare that there is no conflict of interest.

## Supporting information

Supplementary Material

## Data Availability

Sequences were deposited in the NCBI SRA database with under the Bio‐Project PRJNA781104. Data and scripts are available online in the Zenodo repository using https://doi.org/10.5281/zenodo.5710318.
