# Supplementary material for: Fruit fly phylogeny imprints bacterial gut microbiota
Source: Evol Appl. 2022 May 3;15(10):1621–38. doi: 10.1111/eva.13352 (PMC9624087; doi:10.1111/eva.13352)
Supplement: Supplementary file 1 — Supplementary Material [file EVA-15-1621-s001.docx]

# Supplementary material

## Appendix S1: Mock analysis

Mock communities commonly serve as controls in metabarcoding studies. These allow estimating rates of false positive taxa, and relative abundance distortions. Here we provide inferred community profiles on a commercial mock community of eight bacterial species corresponding in 8 different genera in nonequivalent proportions (Mock ZymoBIOMICS, Microbial Community DNA standard, ref. D6306).

#### Sequence production

In three independent experiments, the Zymo mock community was used as a control and was analyzed using the following protocol. About 10ng of DNA was amplified using specific primers that target the 16S rRNA gene (27F 5′-AGAGTTTGGATCMTGGCTCAG-3′; 1492R 5′-GGTTACCTTGTTACGACTT-3'), as well as subsequent specific barcodes using a 16S Barcoding Kit (SQK-RAB204, Oxford Nanopore Technologies). After bead purification for removal of excess primers, amplification products were attached to rapid sequencing adapters before loading on a MinION flow cell for sequencing.

#### Bioinformatics

Basecalling, demultiplexing and chimera removal were performed using Guppy v2.2.3 (<https://community.nanoporetech.com>). Reads were trimmed (only nucleotides between positions 60 and 1460 were kept) and filtered (only sequences longer than 900 pb and above quality score Q10 were kept) (De Coster et al. 2018): only sequences longer than 900 pb and above quality score Q10 were kept, leading to a total of 71698 sequences (ranging from 7912 to 25744 across samples). Taxonomy was assigned by confronting reads to the Silva 138 database (Quast et al. 2013, Yilmaz et al. 2014) using VSEARCH 2020.8.0 (Rognes et al. 2016) embedded in QIIME2 2020.8 (Bolyen et al. 2019), with perc_identity=0.90, max_accepts = 100, max_rejects = 100 and max_hits = ‘all’. A phyloseq object was produced and imported in R (McMurdie and Holmes 2013, R Core Team 2020) for further exploration. The percentage of assignment for the 16 fruit fly samples was 78.1 % at phylum, class, order, and family levels. It dropped to 74.9 % at the genus level and 34.0 % at the species level.

#### Results

Examining mock samples revealed correct identification of mock taxa at the genus level. In particular, Pseudomonas, which had the lowest expected frequency (0.04) was always retrieved at a congruent frequency, ranging from 0.02 to 0.04 across samples. The highest relative abundance of a false positive was 0.0009 and was associated with Enterobacter. Mock community profiling was both very constant across runs and very close to the expectation (Figure S1), with one noticeable exception, *Lactobacillus fermentum*, which relative abundance was consistently underestimated in all samples. This underestimation of Lactobacillus has already been found in a previous study using mock communities for quantifying and characterizing bias introduced in the sample-processing pipeline (Brooks et al. 2015).

Figure S1: Bacterial composition of mock samples


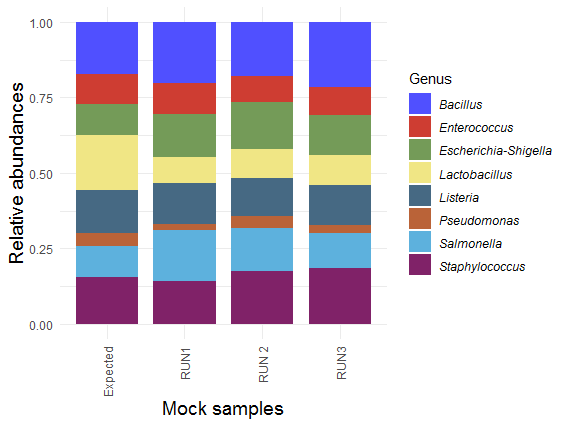


#### Conclusion

All stages along the production of microbiome data may induce errors and biases in inferred community composition (Brooks et al. 2015). Analysis of mock communities can help assess some of these biases and facilitate the interpretation of results from environmental samples. Mock communities can also be used to tune bioinformatics pipelines. For instance, mock communities provide minimal relative abundances of true positive taxa and maximal relative abundances of false positive taxa that can be used as objective criteria to define thresholds to filter contingency tables prior to diversity analyses.

## Appendix S2: Diversity analyses

#### Sample characteristics

Table S1. sample characteristics

| Label | Species | Sample size | Sampling environment | Laboratory generations | Sampling method | Locality | Read count before rarefaction | Sample code |
| --- | --- | --- | --- | --- | --- | --- | --- | --- |
| ZC_Nat | *Z. cucurbitae* | 30 | Nature | - | Traps | Bassin plat | 6335 | RUN1_barcode05 |
| ZC_Lab | *Z. cucurbitae* | 30 | Laboratory | 85 | Mouth aspirator | UMR PVBMT | 4066 | RUN1_barcode01 |
| BD_Nat | *B. dorsalis* | 30 | Nature | - | Traps | Vieux Domaine | 3951 | RUN1_barcode06 |
| BD_Lab | *B. dorsalis* | 30 | Laboratory | 3 | Mouth aspirator | UMR PVBMT | 25483 | RUN1_barcode02 |
| BZ_Nat | *B. zonata* | 30 | Nature | - | Traps | Vieux Domaine | 12753 | RUN1_barcode07 |
| BZ_Lab | *B. zonata* | 30 | Laboratory | 168 | Mouth aspirator | UMR PVBMT | 4657 | RUN1_barcode03 |
| CQ_Nat | *C. quilicii* | 30 | Nature | - | Infested fruits | Cilaos | 3806 | RUN3_barcode05 |
| CQ_Lab | *C. quilicii* | 29 | Laboratory | 23 | Mouth aspirator | UMR PVBMT | 4496 | RUN1_barcode04 |
| DC_Nat | *D. ciliatus* | 30 | Nature | - | Infested fruits | La Saline | 15349 | RUN2C_barcode04 |
| DC_Lab | *D. ciliatus* | 30 | Laboratory | 4 | Mouth aspirator | UMR PVBMT | 3935 | RUN3_barcode01 |
| DD_Nat | *D. demmerezi* | 27 | Nature | - | Infested fruits | Petite île | 16222 | RUN2C_barcode06 |
| DD_Lab | *D. demmerezi* | 30 | Laboratory | 39 | Mouth aspirator | UMR PVBMT | 20906 | RUN2C_barcode05 |
| NC_Nat | *N. cyanescens* | 23 | Nature | - | Infested fruits | Saint-Joseph | 13139 | RUN2C_barcode08 |
| NC_Lab | *N. cyanescens* | 25 | Laboratory | 39 | Mouth aspirator | UMR PVBMT | 30207 | RUN2C_barcode07 |
| CC_Nat | *C. capitata* | 28 | Nature | - | Infested fruits | Saint-Leu | 3250 | RUN3_barcode04 |
| CC_Lab | *C. capitata* | 30 | Laboratory | 46 | Mouth aspirator | UMR PVBMT | 4511 | RUN3_barcode03 |

For each sample, the fruit fly species, the number of individual guts pooled and the number of reads after bioinformatics treatments are provided. Samples from nature were collected in various localities of Reunion island, using either baited traps (*Bactrocera* and *Zeugodacus* species) or sets of infested fruits from various plant species (other fly species). Details about the infested fruits, from which insects were collected: CC_Nat: 20 from orange jasmine (*Murraya paniculata*) and 8 from Chinese quince (*Pseudocydonia sinensis*); CQ_Nat: 13 males from strawberry guava (*Psidium cattleianum*) and 17 from common guava (*Psidium guajava*); DC_Nat: zucchini (*Cucurbita pepo*); DD_Nat: bitter melon (*Momordica* *charantia*); NC_Nat: 12 from bugweed (*Solanum mauritianum*) and 11 from tomato (*Solanum lycopersicum*). Laboratory samples were collected from long-term rearing experiments in PVBMT laboratory in Saint-Pierre, Reunion. The number of generations since the establishment of the corresponding population in the laboratory is provided. Laboratory populations were initially sampled from fruits: BD_Lab and BZ_Lab: Indian almond (*Terminalia catappa*); CC_Lab: beach naupaka (*Scaevola taccada*); CQ_Lab: rose apple ([*Syzygium jambos*](https://en.wikipedia.org/wiki/Syzygium_jambos)); DC_Lab and DD_Lab: cucumber (*Cucumis sativus*); NC_Lab: bugweed (*Solanum mauritianum*); ZC_Lab: zucchini (*Cucurbita pepo*). Laboratory populations were reared on artificial diet. Larvae of the generalist species (*C. capitata*, *C. quilicii*, *B. dorsalis*, and *B. zonata*) were given dehydrated carrot powder, brewer’s yeast, sugar, dehydrated potato, water, Nipagin/Sodium benzoate, HCl (1.65%), agar and wheat germ. The diet was supplemented with fresh zucchini for the two specialists of Cucubitaceae (*D. demmerezi* and *Z. cucurbitae*) and with fresh potato for the specialist of Solanaceae (*N. cyanescens*). Laboratory populations have been occasionally supplemented with individuals caught in the wild.

#### Rarefaction curves and presence-absence of bacterial genera


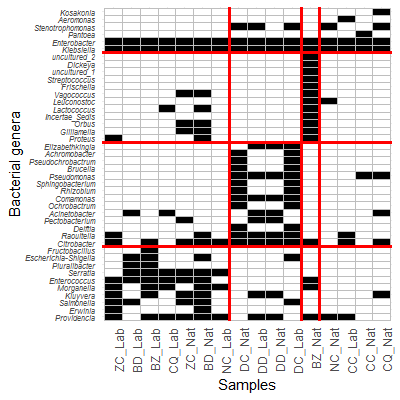

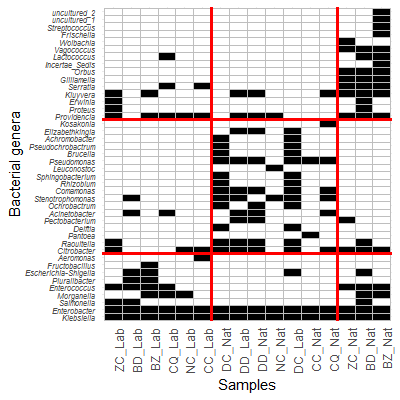
Figure S2: Two random examples of binary matrices. The matrices were obtained by rarefying samples to 3000 reads and applying a 3-read threshold (as described in the main text). Black stands for presence (i.e., above 3 reads after rarefaction), white stands for absence. Red lines delimitate clusters obtained through the leading eigenvalue method as described in the main text. The orders of samples (x-axis) and bacterial genera (y-axis) differ between the two matrices to facilitate cluster visualization. These two examples illustrate the variability on presence-absence data obtained after rarefaction.

Figure S3: Rarefaction curves. Estimates of diversity statistics as a function of the number of reads in samples, as provided by R package iNEXT (Hsieh et al. 2016). Each graph corresponds to one sample. Curves represent measures of Hill numbers of orders 0 (pink curves, richness in numbers of genera), 1 (green curves, exponential of Shannon entropy) and 2 (blue curves, inverse of Simpson concentration). Colored areas around curves are 95% associated confidence intervals. Plain lines represent diversities estimated from rarefaction of actual data. Dotted lines are interpolated estimates. Dots represent actual read numbers in samples. In some samples, species richness did not reach a plateau, indicating that obtaining more reads would have allowed detecting more bacterial genera, *e.g.*, RUN1_barcode01, RUN2C_barcode07, RUN3_barcode03 and others. However, in all samples Shannon and Simpson diversities attained a plateau for read numbers well below the 3000-read threshold used for rarefaction in presence-absence analyses. Hence, unlike genera numbers, Shannon and Simpson alpha diversities could be considered as correctly estimated.


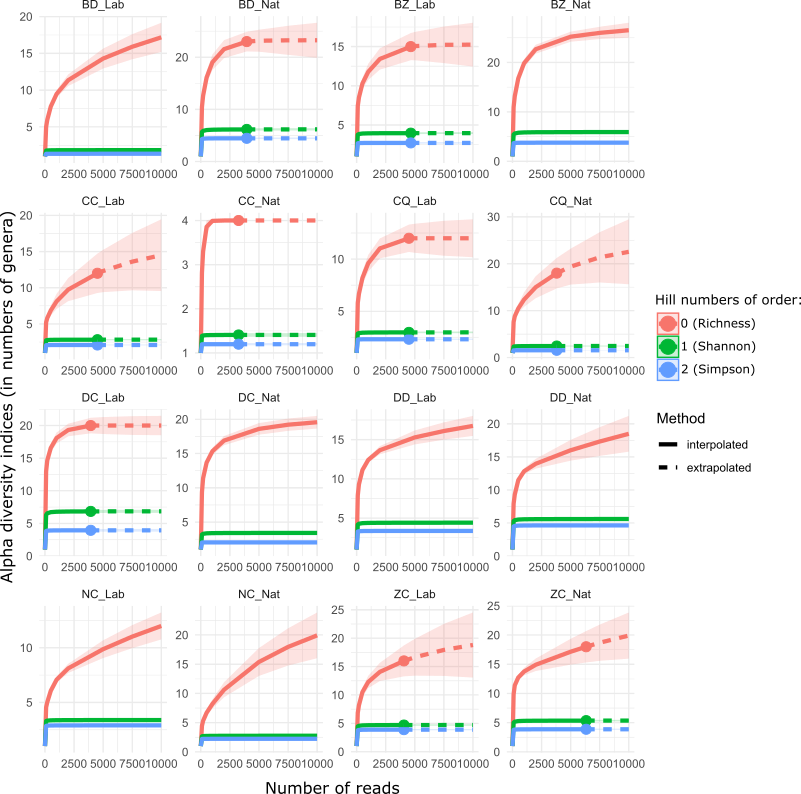


#### Bacterial diversity

Table S2. Full list of bacterial genera identified with their taxonomy.

| Phylum | Class | Order | Family | Genus |
| --- | --- | --- | --- | --- |
| Bacteroidota | Bacteroidia | Flavobacteriales | Weeksellaceae | *Elizabethkingia* |
|  |  | Sphingobacteriales | Sphingobacteriaceae | *Sphingobacterium* |
| Firmicutes | Bacilli | Lactobacillales | Enterococcaceae | *Enterococcus* |
|  |  |  | Lactobacillaceae | *Lactobacillus* |
|  |  |  | Leuconostocaceae | *Fructobacillus* |
|  |  |  |  | *Leuconostoc* |
|  |  |  | Streptococcaceae | *Lactococcus* |
|  |  |  | Streptococcaceae | *Streptococcus* |
|  |  |  | Uncultured_1 | Uncultured_1 |
|  |  |  | Vagococcaceae | *Vagococcus* |
| Proteobacteria | Alphaproteobacteria | Rhizobiales | Rhizobiaceae | *Brucella* |
|  |  |  | Rhizobiaceae | *Ochrobactrum* |
|  |  |  | Rhizobiaceae | *Pseudochrobactrum* |
|  |  |  | Rhizobiaceae | *Rhizobium** |
|  |  | Rickettsiales | Anaplasmataceae | *Wolbachia* |
| Proteobacteria | Gammaproteobacteria | Aeromonadales | Aeromonadaceae | *Aeromonas* |
|  |  | Burkholderiales | Alcaligenaceae | *Achromobacter* |
|  |  |  |  | *Bordetella* |
|  |  |  | Comamonadaceae | *Comamonas* |
|  |  |  |  | *Delftia* |
|  |  |  | Oxalobacteraceae | *Herbaspirillum* |
|  |  | Enterobacterales | Enterobacteriaceae | *Citrobacter* |
|  |  |  |  | *Enterobacter* |
|  |  |  |  | *Escherichia-Shigella* |
|  |  |  |  | *Klebsiella* |
|  |  |  |  | *Kluyvera* |
|  |  |  |  | *Kosakonia* |
|  |  |  |  | *Pluralibacter* |
|  |  |  |  | *Raoultella* |
|  |  |  |  | *Salmonella* |
|  |  |  | Erwiniaceae | *Erwinia* |
|  |  |  |  | *Pantoea* |
|  |  |  | Morganellaceae | *Incertae_Sedis* |
|  |  |  |  | *Morganella* |
|  |  |  |  | *Proteus* |
|  |  |  |  | *Providencia* |
|  |  |  |  | Uncultured_2 |
|  |  |  | Pectobacteriaceae | *Dickeya* |
|  |  |  |  | *Pectobacterium* |
|  |  |  | Yersiniaceae | *Serratia* |
|  |  | Orbales | Orbaceae | *Frischella* |
|  |  |  |  | *Gilliamella* |
|  |  |  |  | *Orbus* |
|  |  | Pseudomonadales | Moraxellaceae | *Acinetobacter* |
|  |  |  | Pseudomonadaceae | *Pseudomonas* |
|  |  | Xanthomonadales | Xanthomonadaceae | *Stenotrophomonas* |

* The Silva database does not distinguish between the genera Allorhizobium-Neorhizobium-Pararhizobium-Rhizobium.

Table S3. The weighted incidence matrix

| Bacterial genus | ZC_Lab | BD_Lab | BZ_Lab | CQ_Lab | ZC_Nat | BD_Nat | BZ_Nat | DC_Nat | DD_Lab | DD_Nat | NC_Lab | NC_Nat | DC_Lab | CC_Lab | CC_Nat | CQ_Nat |
| --- | --- | --- | --- | --- | --- | --- | --- | --- | --- | --- | --- | --- | --- | --- | --- | --- |
| *Elizabethkingia* | 0 | 0 | 0 | 0 | 0 | 0 | 0 | 15 | 104 | 55 | 0 | 0 | 38 | 0 | 0 | 0 |
| *Sphingobacterium* | 0 | 1 | 0 | 0 | 0 | 0 | 0 | 160 | 0 | 9 | 0 | 0 | 108 | 0 | 0 | 0 |
| *Enterococcus* | 25 | 1007 | 412 | 9 | 62 | 72 | 294 | 0 | 0 | 0 | 3 | 0 | 0 | 0 | 0 | 1 |
| *Lactobacillus* | 0 | 2 | 0 | 0 | 1 | 0 | 13 | 0 | 0 | 0 | 0 | 0 | 0 | 0 | 0 | 0 |
| *Fructobacillus* | 0 | 0 | 22 | 0 | 0 | 0 | 2 | 0 | 0 | 0 | 0 | 0 | 0 | 0 | 0 | 0 |
| *Leuconostoc* | 0 | 0 | 5 | 0 | 0 | 0 | 12 | 0 | 0 | 0 | 0 | 102 | 0 | 0 | 0 | 0 |
| *Lactococcus* | 0 | 1 | 0 | 99 | 0 | 130 | 737 | 0 | 0 | 0 | 32 | 4 | 0 | 0 | 0 | 1 |
| *Streptococcus* | 0 | 0 | 0 | 0 | 0 | 3 | 45 | 0 | 0 | 0 | 0 | 2 | 0 | 0 | 0 | 0 |
| *uncultured_1* | 0 | 0 | 0 | 0 | 0 | 0 | 135 | 0 | 0 | 0 | 0 | 0 | 0 | 0 | 0 | 0 |
| *Vagococcus* | 0 | 0 | 0 | 0 | 54 | 45 | 105 | 0 | 0 | 0 | 0 | 0 | 0 | 0 | 0 | 0 |
| *Brucella* | 0 | 0 | 0 | 0 | 0 | 0 | 0 | 32 | 1 | 2 | 1 | 3 | 3 | 0 | 0 | 0 |
| *Ochrobactrum* | 0 | 1 | 0 | 0 | 0 | 0 | 0 | 294 | 6 | 28 | 0 | 5 | 150 | 0 | 0 | 1 |
| *Pseudochrobactrum* | 0 | 0 | 0 | 0 | 0 | 0 | 0 | 40 | 0 | 0 | 0 | 0 | 68 | 0 | 0 | 0 |
| *Rhizobium* | 0 | 1 | 0 | 0 | 0 | 0 | 0 | 239 | 0 | 1 | 0 | 3 | 190 | 0 | 0 | 1 |
| *Wolbachia* | 0 | 0 | 0 | 0 | 11 | 0 | 0 | 0 | 0 | 0 | 0 | 0 | 0 | 0 | 0 | 0 |
| *Aeromonas* | 0 | 0 | 0 | 0 | 0 | 0 | 0 | 0 | 1 | 0 | 0 | 0 | 0 | 81 | 0 | 0 |
| *Achromobacter* | 0 | 0 | 0 | 0 | 0 | 0 | 0 | 10 | 0 | 1 | 0 | 1 | 79 | 0 | 0 | 0 |
| *Bordetella* | 0 | 0 | 0 | 0 | 0 | 0 | 0 | 6 | 0 | 0 | 0 | 0 | 6 | 0 | 0 | 0 |
| *Comamonas* | 0 | 6 | 0 | 0 | 0 | 0 | 0 | 52 | 142 | 77 | 0 | 0 | 571 | 0 | 0 | 4 |
| *Delftia* | 1 | 2 | 0 | 0 | 0 | 1 | 0 | 127 | 0 | 1 | 0 | 0 | 254 | 0 | 0 | 3 |
| *Herbaspirillum* | 0 | 0 | 0 | 0 | 0 | 0 | 0 | 0 | 0 | 0 | 0 | 0 | 0 | 0 | 0 | 6 |
| *Citrobacter* | 349 | 22 | 4 | 3 | 2036 | 718 | 958 | 248 | 7721 | 5331 | 6544 | 1 | 5 | 765 | 0 | 66 |
| *Enterobacter* | 1460 | 21812 | 2613 | 2590 | 671 | 419 | 5790 | 458 | 198 | 1731 | 1713 | 3168 | 146 | 2966 | 2958 | 3026 |
| *Escherichia-Shigella* | 0 | 108 | 29 | 3 | 0 | 7 | 6 | 1 | 0 | 1 | 3 | 10 | 22 | 0 | 0 | 2 |
| *Klebsiella* | 1177 | 1689 | 280 | 21 | 2312 | 1462 | 414 | 10499 | 2213 | 3755 | 64 | 1711 | 1825 | 548 | 258 | 149 |
| *Kluyvera* | 13 | 0 | 15 | 2 | 95 | 16 | 34 | 0 | 51 | 49 | 3 | 1 | 1 | 2 | 0 | 197 |
| *Kosakonia* | 0 | 0 | 0 | 0 | 0 | 0 | 0 | 0 | 0 | 0 | 0 | 1 | 0 | 0 | 0 | 164 |
| *Pluralibacter* | 1 | 662 | 199 | 0 | 0 | 0 | 0 | 0 | 0 | 0 | 0 | 0 | 0 | 1 | 0 | 0 |
| *Raoultella* | 254 | 0 | 0 | 0 | 0 | 4 | 1 | 1899 | 7901 | 2774 | 2 | 1 | 53 | 4 | 0 | 0 |
| *Salmonella* | 6 | 66 | 2 | 0 | 12 | 12 | 6 | 0 | 0 | 0 | 0 | 1 | 6 | 1 | 0 | 0 |
| *Erwinia* | 17 | 0 | 1 | 0 | 0 | 10 | 0 | 0 | 0 | 0 | 0 | 0 | 0 | 0 | 0 | 0 |
| *Pantoea* | 1 | 3 | 0 | 0 | 1 | 3 | 1 | 0 | 1 | 1 | 2 | 8 | 0 | 3 | 13 | 2 |
| *Incertae_Sedis* | 0 | 1 | 0 | 0 | 1 | 0 | 27 | 0 | 0 | 0 | 0 | 0 | 0 | 0 | 0 | 0 |
| *Morganella* | 4 | 0 | 152 | 164 | 0 | 9 | 50 | 3 | 12 | 3 | 14895 | 1 | 0 | 0 | 0 | 0 |
| *Proteus* | 16 | 0 | 0 | 0 | 1 | 15 | 9 | 0 | 0 | 0 | 0 | 0 | 0 | 0 | 0 | 0 |
| *Providencia* | 736 | 2 | 918 | 198 | 577 | 100 | 367 | 3 | 1517 | 1771 | 6874 | 8001 | 6 | 130 | 0 | 0 |
| *uncultured_2* | 0 | 0 | 0 | 0 | 0 | 0 | 13 | 0 | 0 | 0 | 0 | 0 | 0 | 0 | 0 | 0 |
| *Dickeya* | 0 | 0 | 0 | 0 | 0 | 0 | 13 | 0 | 0 | 0 | 0 | 0 | 0 | 0 | 0 | 0 |
| *Pectobacterium* | 2 | 0 | 0 | 0 | 108 | 3 | 5 | 0 | 35 | 89 | 1 | 0 | 0 | 0 | 0 | 0 |
| *Serratia* | 4 | 30 | 3 | 1397 | 112 | 5 | 17 | 16 | 3 | 0 | 65 | 0 | 0 | 9 | 0 | 1 |
| *Frischella* | 0 | 0 | 0 | 0 | 3 | 2 | 19 | 0 | 0 | 0 | 0 | 0 | 0 | 0 | 0 | 0 |
| *Gilliamella* | 0 | 7 | 0 | 0 | 132 | 788 | 2539 | 0 | 0 | 0 | 0 | 4 | 0 | 0 | 0 | 0 |
| *Orbus* | 0 | 2 | 0 | 0 | 146 | 125 | 1141 | 0 | 1 | 0 | 0 | 2 | 0 | 0 | 0 | 0 |
| *Acinetobacter* | 0 | 47 | 0 | 8 | 0 | 0 | 0 | 0 | 455 | 63 | 4 | 0 | 0 | 1 | 0 | 58 |
| *Pseudomonas* | 0 | 0 | 0 | 2 | 0 | 2 | 0 | 1060 | 522 | 479 | 1 | 1 | 302 | 0 | 21 | 29 |
| *Stenotrophomonas* | 0 | 11 | 2 | 0 | 0 | 0 | 0 | 187 | 22 | 1 | 0 | 108 | 102 | 0 | 0 | 95 |

The weighted incidence matrix contains read counts observed after bioinformatic treatments (and before rarefaction) for each of 46 bacterial genera in each of 16 samples.

Figure S4: Relationships between alpha diversity of laboratory samples and the number of generations in the laboratory. Alpha diversity, estimated as a Hill number of order 1 (exponential of Shannon diversity, in genus equivalents), does not correlate with the number of generations that laboratory populations have spent in the laboratory.

Figure S5: Ordination on presence-absence matrices using NMDS on Jaccard distances. Here are shown NMDS ordinations from three random presence-absence matrices. Legends are identical to Figure 3 (colors depict fly phylogenetic group, triangles stand for field samples and circles for laboratory samples). As expected, results show some variability. However, the main message that samples ordinate according to fly phylogeny, rather than sampling environment or specialization, holds whatever the matrix, as well as with the weighted incidence matrix which ordination is shown in Figure 3.

1. NMDS stress value : 0.156

1. NMDS stress value : 0.115

1. NMDS stress value : 0.188

#### Characteristics of the 1000 observed binary matrices

Figure S6: Distribution of the number of clusters across the 1000 observed binary matrices. The number of clusters were obtained by the leading eigenvalue method, as described in the main text.


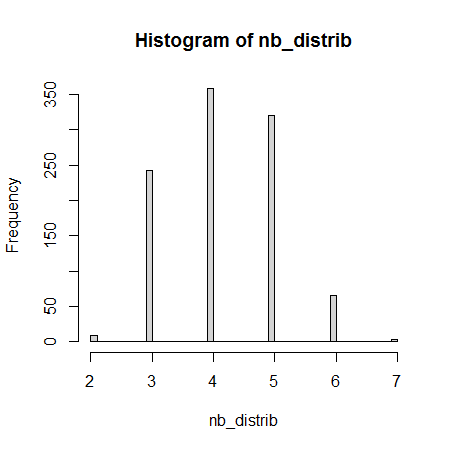


Number of clusters

Figure S7: Distribution of modularity across observed binary matrices with its significance. Left panel: Distribution of modularity in observed binary matrices (in dark grey) and in simulated random matrices (in light grey). The *p*-value of the average observed modularity among simulated matrices is 0.002. Right panel: The distribution of *p*-values of observed modularity values among corresponding simulated matrices.


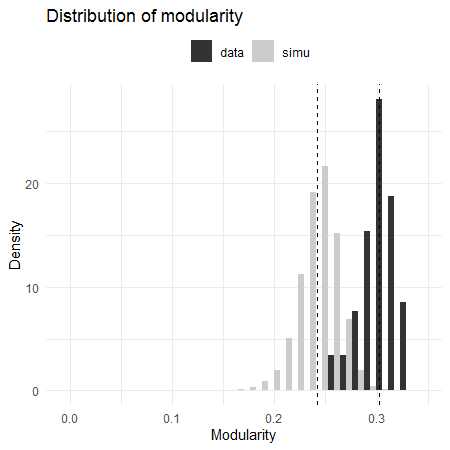


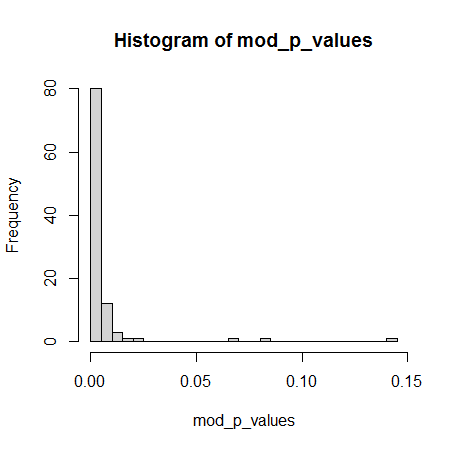


p-value of modularity

#### Latent block model (LBM) clustering of the weighted incidence matrix

Table S4: Competing LBM models ranked by their ICL for the 16-sample dataset. Models differ by the number of samples and taxa blocks they assume. Different numbers of blocks imply different numbers of parameters.

| Total number of blocks | Number of parameters | Number of sample blocks | Number of taxa blocks | ICL | Log-likelihood |
| --- | --- | --- | --- | --- | --- |
| 6 | 16 | 3 | 3 | -842.8870 | -796.8322 |
| 7 | 20 | 3 | 4 | -843.1267 | -785.2997 |
| 9 | 30 | 4 | 5 | -847.1590 | -759.7254 |
| 8 | 25 | 4 | 4 | -848.0879 | -775.7161 |
| 5 | 12 | 2 | 3 | -850.4894 | -815.6898 |
| 4 | 9 | 2 | 2 | -874.2060 | -847.8890 |
| 2 | 4 | 1 | 1 | -891.0615 | -877.9030 |
| 3 | 6 | 2 | 1 | -898.9323 | -881.0978 |

Figure S8: Distribution of ICL values for the LBM models explored on the 16-sample dataset. Dots represent ICL values of individual models tested during the optimization algorithm. Red dots are the best models for each pair of number of sample blocks (Q1) and number of taxa blocks explored (Q2). The best model had Q1 = 3 blocks of samples and Q2 = 4 blocks of taxa, as depicted in Figure 5 of the manuscript.


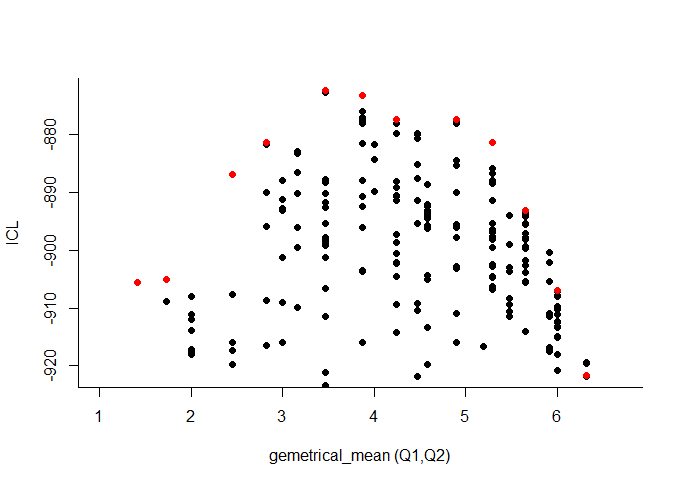


Geometrical mean of Q1 and Q2

Figure S9: Sub-communities identified using LBM clustering on read count matrices for laboratory (upper panel) and field (lower panel) samples separately. Log-transformed read counts are represented on a continuous gradient from white for log10(reads+1) = 0, to black for log10(reads+1) = 4.4. Red lines delimit clusters identified under the best latent block model. Dendograms along the y-axis represent fly phylogeny (branch length do not represent divergence). For field samples, sample groups defined according to their gut microbiota correspond to fly tribes (Dacinae vs. Ceratitinae).

#### References cited in supplementary material

Bolyen E. et al. (2019) Reproducible, interactive, scalable and extensible microbiome data science using QIIME 2. Nature Biotechnology 37: 852-857.

Brooks J.P. et al. (2015) The truth about metagenomics: quantifying and counteracting bias in 16S rRNA studies. BMC Microbiology 15:66.

De Coster W., D’Hert S., Schultz D.T., Cruts M. & Van Broeckhovn C. (2018) NanoPack: visualizing and processing long-read sequencing data. Bioinformatics 34: 2666-2669.

Hsieh T. C., Ma K. H. & Chao A. (2016). iNEXT: an R package for rarefaction and extrapolation of species diversity (H ill numbers). Methods in Ecology and Evolution 7: 1451-1456.

McMurdie P.J. & Holmes S. (2013) phyloseq: An R package for reproducible interactive analysis and graphics of microbiome census data. PLOS ONE 8: e61217.

Quast C. et al. (2013) The SILVA ribosomal RNA gene database project: improved data processing and web-based tools. Nucleic Acids Research 41: D590-D596.

Rognes T., Flouri T., Nichols B., Quince C. & Mahe F. (2016) VSEARCH: a versatile open source tool for metagenomics. PEER J 4: e2584.

Yilmaz P. (2014) The SILVA and “All-species Living Tree Project (LTP)” taxonomic frameworks. Nucleic Acids Research 42: D643-D648.
